# β Cell Gαs signaling is critical for physiological and pharmacological enhancement of insulin secretion

**DOI:** 10.1172/JCI183741

**Published:** 2025-06-17

**Authors:** Megan E. Capozzi, David Bouslov, Ashot Sargsyan, Michelle Y. Chan, Alex Chen, Sarah M. Gray, Katrina Viloria, Akshay Bareja, Jonathan D. Douros, Sophie L. Lewandowski, Jason C.L. Tong, Annie Hasib, Federica Cuozzo, Elizabeth C. Ross, Matthew W. Foster, Lee S. Weinstein, Mehboob A. Hussain, Matthew J. Merrins, Francis S. Willard, Mark O. Huising, Kyle W. Sloop, David J. Hodson, David A. D’Alessio, Jonathan E. Campbell

**Affiliations:** 1 Duke Molecular Physiology Institute, Duke University, Durham, North Carolina, USA.; 2 Department of Medicine, Division of Metabolism, Endocrinology, and Nutrition, University of Washington, Seattle, Washington, USA.; 3 Department of Neurobiology, Physiology and Behavior, University of California, Davis, Davis, California, USA.; 4 Oxford Centre for Diabetes, Endocrinology and Metabolism, NIHR Oxford Biomedical Research Centre, Churchill Hospital, Radcliffe Department of Medicine, University of Oxford, Oxford, United Kingdom.; 5 Department of Metabolism and Systems Science, College of Medicine and Health, University of Birmingham, Birmingham, United Kingdom.; 6 Indiana Biosciences Research Institute, Indianapolis, Indiana, USA.; 7 Department of Medicine, Division of Endocrinology, Diabetes and Metabolism, University of Wisconsin–Madison, Madison, Wisconsin, USA.; 8 Proteomics and Metabolomics Core Facility, Duke University, Durham, North Carolina, USA.; 9 Metabolic Diseases Branch, National Institute of Diabetes, Digestive, and Kidney Diseases, NIH, Bethesda, Maryland, USA.; 10 Department of Internal Medicine, University of California, Irvine, California, USA.; 11 Molecular Pharmacology, Lilly Research Laboratories, Eli Lilly and Company, Indianapolis, Indiana, USA.; 12 Department of Physiology and Membrane Biology, School of Medicine, University of California, Davis, Davis, California, USA.; 13 Diabetes, Obesity and Complications, Lilly Research Laboratories, Eli Lilly and Company, Indianapolis, Indiana, USA.; 14 Department of Medicine and; 15 Department of Pharmacology and Cancer Biology, Duke University, Durham, North Carolina, USA.

**Keywords:** Endocrinology, Metabolism, Diabetes, G proteins, Insulin

## Abstract

The incretin peptides glucose-dependent insulinotropic polypeptide and glucagon-like peptide-1 receptors coordinate β cell secretion that is proportional to nutrient intake. This effect permits consistent and restricted glucose excursions across a range of carbohydrate intake. The canonical signaling downstream of ligand-activated incretin receptors involves coupling to Gαs protein and generation of intracellular cAMP. However, recent reports have highlighted the importance of additional signaling nodes engaged by incretin receptors, including other G proteins and β-arrestin proteins. Here, the importance of Gαs signaling was tested in mice with conditional, postdevelopmental β cell deletion of *Gnas* (encoding Gαs) under physiological and pharmacological conditions. Deletion of Gαs/cAMP signaling induced immediate and profound hyperglycemia that responded minimally to incretin receptor agonists, a sulfonylurea, or bethanechol. While islet area and insulin content were not affected in *Gnas*^βcell–/–^, perifusion of isolated islets demonstrated impaired responses to glucose, incretins, acetylcholine, and IBMX In the absence of Gαs, incretin-stimulated insulin secretion was impaired but not absent, with some contribution from Gαq signaling. Collectively, these findings validate a central role for cAMP in mediating incretin signaling, but also demonstrate broad impairment of insulin secretion in the absence of Gαs that causes both fasting hyperglycemia and glucose intolerance.

## Abstract 2

The Gs/cAMP pathway is indepensible in beta-cells for the activity of all insulin secretegogues, including incretin receptor agonists, other GPCRs, and sulfonylureas.

## Introduction

The control of postprandial metabolism incorporates multiple complex processes to efficiently coordinate the absorption, tissue distribution, cellular uptake, and metabolism of ingested nutrients. Central to this process is secretion of appropriate levels of insulin from pancreatic β cells. Insufficient insulin secretion resulting from either loss of β cell mass in type 1 diabetes or β cell dysfunction in type 2 diabetes (T2D) manifests as hyperglycemia and contributes to increased morbidity and mortality (1). Inappropriate insulin hypersecretion is also detrimental, as it can cause hypoglycemia in the extreme; in lesser amounts, it has been linked to obesity, insulin resistance, and hepatic steatosis (2, 3). Thus, proper β cell function requires the integration of a range of signals elicited by feeding that create a level of insulinemia carefully matched to nutrient load. A complete understanding of coordinate regulation of insulin secretion, including a role for the central nervous system (4), is currently lacking, a gap reflected by ongoing debate over the relative importance of various β cell stimuli (5). However, taken together, it is clear that β cells respond to inputs from multiple sources with very precise insulin secretion in healthy individuals. Nonetheless, this complex system also provides broad opportunity for failure. This is exemplified by the difficulty in identifying the mechanisms of β cell dysfunction in diabetes to simple lesions that are amenable to straightforward solutions.

Our group has recently defined an axis that governs insulin secretion termed α–to–β cell communication. We (6–8), and others (9–11), have expanded on the original observations shown decades prior (12) to describe how proglucagon peptides from α cells are essential for β cell function. α Cells produce both glucagon- and glucagon-like peptide 1–related (GLP-1–related) peptides, both of which agonize the GLP-1 receptor (GLP-1R). Although the relative importance of these 2 peptides remains unresolved, they are responsible for approximately 50%–80% of insulin secretion from isolated rodent or human islets (6, 7). We found that both pharmacological or genetic interruption of α–to–β cell communication greatly reduced β cell cAMP levels, accounting for severely impaired insulin secretion in response to glucose and a range of other stimuli, which could be corrected by restoring cAMP levels (6). In vivo, genetic, or pharmacological disruption of the GLP-1R causes glucose intolerance in part due to impaired insulin secretion (7, 13, 14). Moreover, these findings have taken on clinical importance as the GLP-1R has been effectively targeted to lower glucose in T2D, either alone or when coupled with agents that also agonize the glucose-dependent insulinotropic polypeptide receptor (GIPR) (15).

The current consensus is that activation of the GLP-1R and GIPR generates cAMP as the principle secondary messenger that drives intracellular signaling cascades important for insulin biosynthesis and secretion (16). Mechanistically, both incretin receptors couple to and activate Gαs, enhance production of cAMP through adenylyl cyclase, and engage PKA and other downstream signaling pathways to promote insulin secretion. In addition, it has become apparent that both incretin receptors also couple with additional signaling nodes, including Gαq and β-arrestin isoforms (17–19), potential alternative signaling pathways that also affect β cell function. In particular, it has been proposed that the GLP-1R utilizes Gαq rather than Gαs under specific circumstances. One group indicated that physiological concentrations of GLP-1 (1–10 pM) stimulate insulin secretion in part through a Gαs/cAMP/PKA pathway, but also rely on Gαq activation to drive Ca^2+^ via PKC (20). Another study reported that chronic hyperglycemia, mimicking T2D, induced the GLP-1R to switch from Gαs to Gαq as the proximal step leading to increased insulin secretion (21). Indeed, this switch was indicated to be specific for the GLP-1R, and not observed with the GIPR, demonstrating a plasticity that allowed the GLP-1R to continue functioning in a glucotoxic environment. The authors proposed that this observation explains why GLP-1R retains insulinotropic action in the setting of T2D, while the actions of GIPR are lost (21). A provocative hypothesis stemming from these results is that the Gαs/cAMP pathway may not be as critical as previously thought for insulin secretion during physiological activation of the GLP-1R or in pharmacological applications. Resolving this question has direct implications for the development of glucose-lowering strategies that target incretin receptors in β cells, particularly in the context of T2D and/or obesity, where pathway selectivity might be altered. To directly test the importance of β cell Gαs signaling, mice were generated with conditional deletion of the β cell *Gnas* gene (which encodes Gαs) to study effects on insulin secretion and glucose tolerance under physiological states and in response to incretin pharmacology.

## Results

Other investigators have previously made mouse models in which *Gnas* was deleted in β cells using Cre recombinase expression driven by the promoters for *Ins1*, *Pdx1*, *Ngn3*, and *Rip* genes. However, Cre recombinase drivers have subsequently been demonstrated to have off-target activity in CNS regions that regulate metabolism or affect gene function during critical developmental windows (22–24). Cardinal features of each of these mouse lines were reduced body weight, decreased β cell mass, and glucose intolerance, which prevented the roles of Gαs in β cell development and β cell function to be distinguished. Moreover, the effects of deleting Gαs signaling on GPCR activity were not directly tested. To address these issues, a β cell–specific, conditional model was made by crossing mice with floxed *Gnas* alleles (*Gnas*^fl/fl^) (25) with a line expressing tamoxifen-inducible *Cre* recombinase under regulation of the mouse insulin promoter (*MIP-Cre^ERT^*) (26); this line was compared with WT littermate controls. Islets from *MIP-Cre^ERT^* mice had moderately increased insulin secretion compared with controls (Supplemental Figure 1A; supplemental material available online with this article; https://doi.org/10.1172/JCI183741DS1). However, glucose tolerance during a mixed-meal tolerance test or in response to i.p. glucose and exogenous incretin agonists was similar between WT and *MIP-Cre^ERT^* mice (Supplemental Figure 1, B–D).

**Figure 1:**
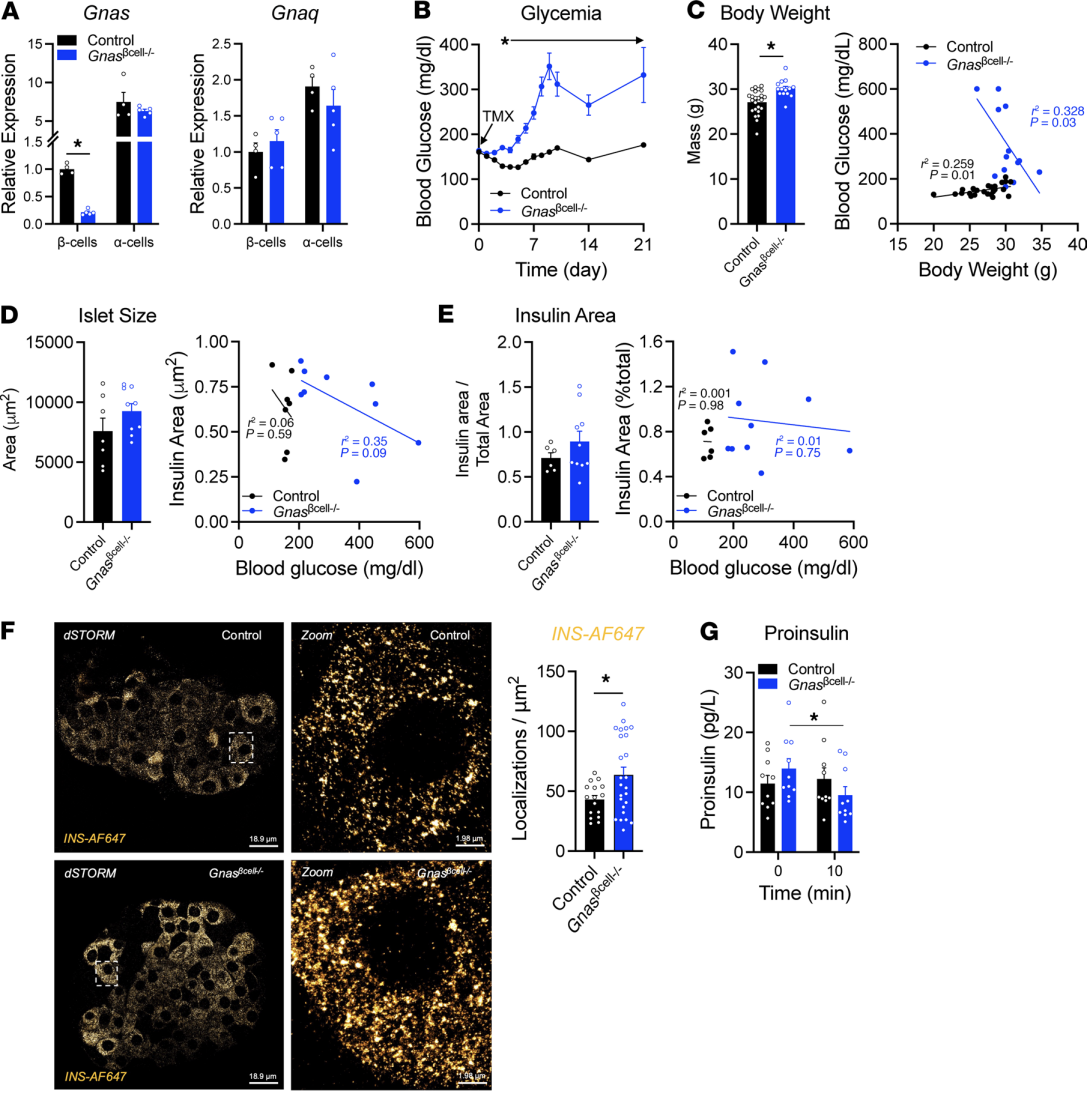
Characterization of *Gnas*^βcell–/–^ mouse islets. (**A**) *Gnas* and *Gnaq* expression in β cell– and α cell–enriched populations (*n* = 4–5). (**B**) Ambient fed glycemia over time in 6- to 8-week-old control (*n* = 29) and *Gnas*^βcell–/–^ (*n* = 23) mice at start of tamoxifen delivery (day 0). (**C**) Body weight of control (*n* = 24) and *Gnas*^βcell–/–^ (*n* = 15) mice and its correlation with fed glycemia. (**D**) Average islet size and its correlation with blood glucose at the time of sacrifice in control (*n* = 7) and *Gnas*^βcell–/–^ (*n* = 9) mice and its correlation with fed glycemia. (**E**) Insulin-positive area per total pancreas area in control (*n* = 7) and *Gnas*^βcell–/–^ (*n* = 9) mice. (**F**) Insulin granule number (localizations/μm^2^) from control and *Gnas*^βcell–/–^ mice, with representative images of insulin granules (*n* = 41 cells from 3 mice per group). Dashed box represents the area selected for zoom, shown in right-hand panel. Scale bars: 18.9 μm (left panels), 1.98 μm (right panels). (**G**) Proinsulin levels at baseline (*t* = 0) and 10 minutes after meal challenge with Ensure (*t* = 10). Data are shown as mean ± SEM, *P < 0.05 as indicated. Data were analyzed by 2-tailed Student’s *t* test (**A** and **C**–**G**), 2-way ANOVA (**B** and **G**), or linear regression (**C**–**E**). dSTORM, direct stochastic optical reconstruction microscopy.

*MIP-Cre^ERT^/Gnas*^fl/fl^ mice were given tamoxifen at 6–8 weeks of age to delete *Gnas* specifically in β cells of young adult animals (*Gnas*^βcell–/–^ mice) (Figure 1A); control mice were treated with an identical tamoxifen protocol. The goal of treatment at 6–8 weeks was to avoid impairment of a critical stage of islet development in preweaned animals as has been previously reported with constitutively active Cre models (22–24). Induction of *Gnas* knockdown with tamoxifen induction of Cre expression led to an immediate rise in glycemia that differed significantly from controls (*Gnas*^fl/fl^) within 72 hours. Blood glucose rose steadily in the *Gnas*^βcell–/–^ mice to stable levels of 15–30 mM (Figure 1B). In contrast to previous reports that deletion of *Gnas* during development decreases growth and limits β cell mass, no deficits in body weight, islet size, or overall β cell mass were noted after *Gnas* was deleted (Figure 1, C–E). We also induced gene knockout in mice starting at 20–24 weeks of age to further ensure the phenotype observed was not attributed to changes in development. In older mice, *Gnas*^βcell–/–^ mice showed a similar rise in glycemia compared with controls, along with equivalent islet number and β cell area (Supplemental Figure 2, A–D) Food intake in the *Gnas*^βcell–/–^ mice was higher than controls and correlated with the degree of hyperglycemia (Supplemental Figure 2E), likely reflective of energy lost through glucosuria (data not shown). The *Gnas*^βcell–/–^ mice had an increase in the number of membrane-resident insulin granules per β cell, as well as larger insulin granules (Figure 1F and Supplemental Figure 2F). This finding supports an increase in insulin biosynthesis, a decrease in insulin granule release, or both. However, there were no major differences in circulating proinsulin levels under fasting or meal-stimulated conditions (Figure 1G), suggesting that insulin processing was similar in control and knockout animals. Consistent with this observation, total insulin content was similar between control and *Gnas*^βcell–/–^ islets (Supplemental Figure 2G). Together, these findings suggest that neither reductions in total β cell numbers nor defects in insulin synthesis or processing are major contributors to the diabetes that develops in *Gnas* knockouts.

**Figure 2:**
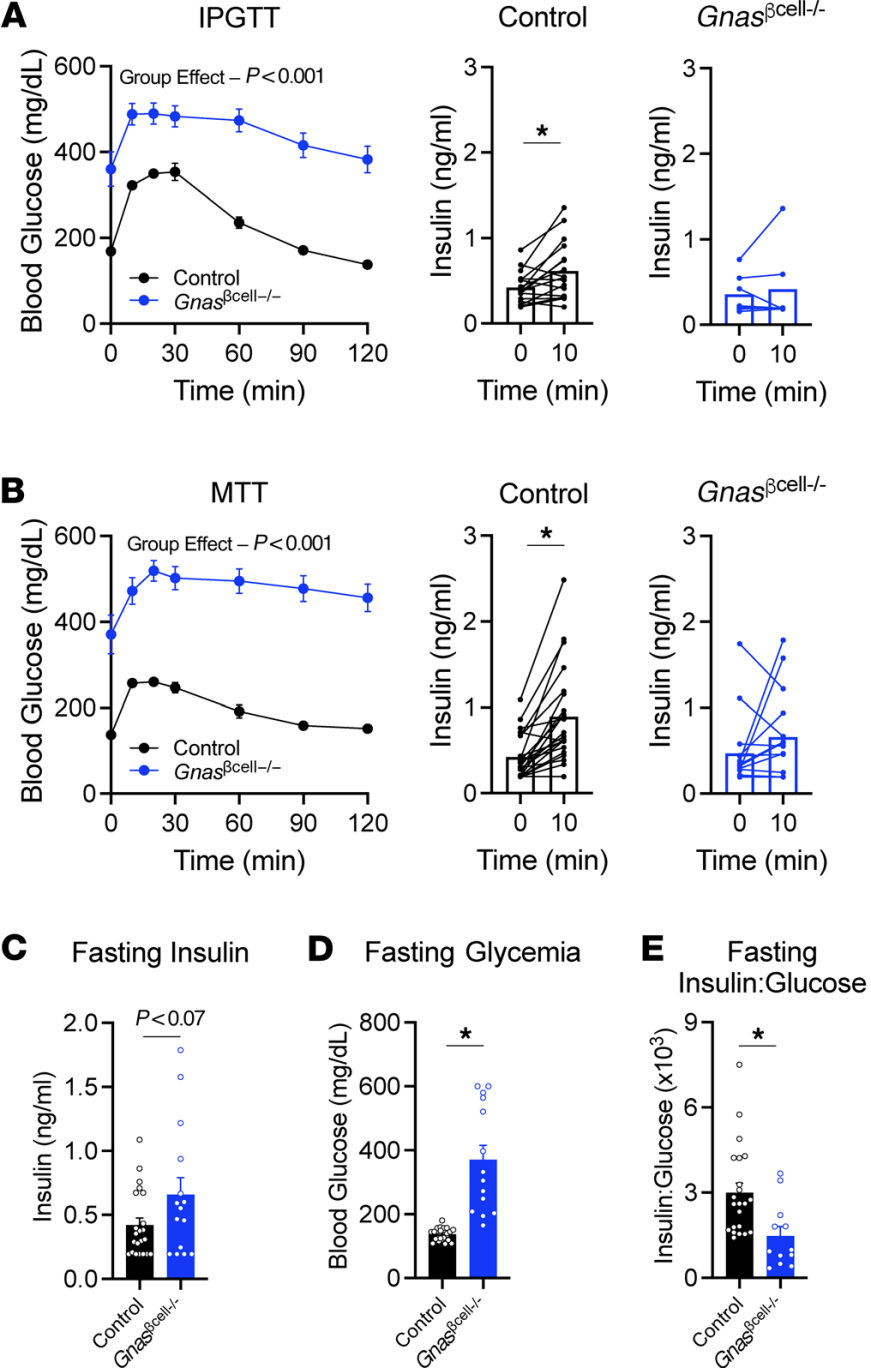
*Gnas*^βcell–/–^ mice are hyperglycemic and do not secrete insulin in response to glucose or meal challenge. (**A**) Intraperitoneal glucose tolerance test (IPGTT) (1.5 g/kg) in control (*n* = 24) and *Gnas*^βcell–/–^ mice (*n* = 15) and insulin at baseline (*t* = 0) and 10 minutes after injection (*t* = 10) in control (*n* = 20) and *Gnas*^βcell–/–^ mice (*n* = 13). (**B**) Mixed-meal tolerance test with Ensure (10 μL/g) in control (*n* = 23) and *Gnas*^βcell–/–^ mice (*n* = 15) and insulin at baseline (*t* = 0) and 10 minutes after injection (*t* = 10) in control (*n* = 23) and *Gnas*^βcell–/–^ mice (*n* = 15). (**C**) Insulin levels in 5-hour-fasted mice, (**D**) glycemia after 5-hour fast, and (**E**) the insulin/glucose ratio for 5-hour-fasted mice (*n* = 22, 15). Data are shown as mean ± SEM, *P < 0.05 as indicated. Data were analyzed by 2-way ANOVA of glycemia data (**A** and **B**) or 2-tailed Student’s *t* test (**A**–**E**).

To assess in vivo glycemic regulation, mice were challenged with i.p. glucose (Figure 2A) or mixed-meal tolerance tests (Figure 2B), 2 physiological interventions that are highly dependent on incretin receptor activity in β cells (6–8, 10, 11, 14). In both settings, the *Gnas*^βcell–/–^ mice displayed severe glucose intolerance and a failure to increase circulating insulin in response to experimental hyperglycemia. These findings were also evident in the fasted state as levels of insulin 5 hours after food was removed were comparable between control and knockout mice despite substantial differences in fasting glycemia (Figure 2, C and D), seen clearly in the insulin/glucose ratios (Figure 2E). To determine if metabolic stress induced by high-fat diet (HFD) would impact the phenotype of *Gnas*^βcell–/–^ mice, we placed a group of mice on 45% kcal from fat diet concurrent with tamoxifen treatment. The *Gnas*^βcell–/–^ mice displayed ambient hyperglycemia on HFD following tamoxifen treatment and elevated fed glycemia along with reduced plasma insulin levels (Supplemental Figure 3, A and B). Interestingly, the impaired β cell function did not impact weight gain on HFD (Supplemental Figure 3C).

**Figure 3:**
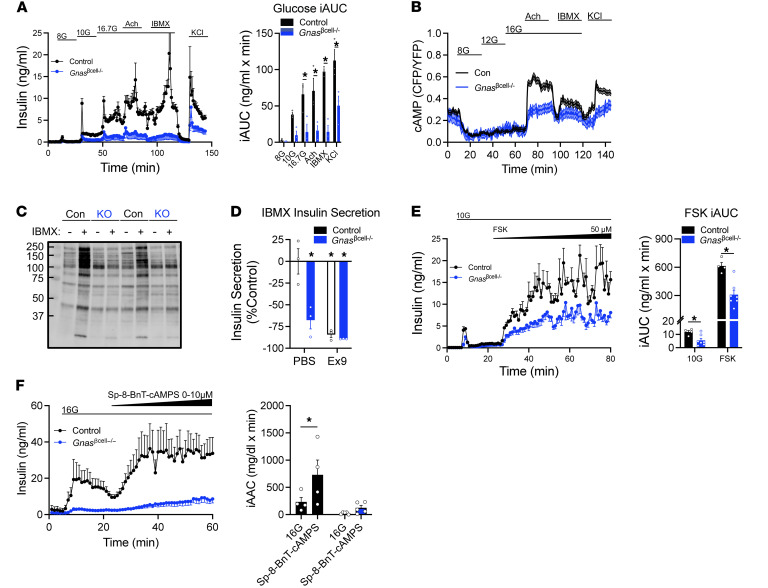
*Gnas*^βcell–/–^ islets have an impaired insulin secretory response to both cAMP-dependent and -independent stimulation. (**A**) Insulin secretion from control or *Gnas*^βcell–/–^ islets in response to increasing glucose doses, acetylcholine (Ach; 10 nM), IBMX (100 μM), and KCl (30 mM) and iAUC of each treatment (*n* = 3). (**B**) cAMP traces in control and *Gnas*^βcell–/–^ islets (*n* = 55, 34). (**C**) Representative blot of pPKA substrates in control- or IBMX-treated islets from control or *Gnas*^βcell–/–^ mice. (**D**) The independent and combined effects of *Gnas* deletion or treatment with Ex9 on insulin secretion in response to an IBMX ramp (*n* = 3). (**E**) Insulin secretion from an FSK ramp in control (*n* = 4) or *Gnas*^βcell–/–^ (*n* = 7) islets and iAUC of each treatment (*n* = 3). (**F**) Insulin secretion in control and *Gnas*^βcell–/–^ islets in response to ramping concentrations of Sp-8-BnT-cAMPS (*n* = 4, 5). Data are shown as mean ± SEM, *P < 0.05 as indicated. Data were analyzed by 2-way ANOVA of iAUC (**A** and **D**–**F**).

To explore the role of Gαs in insulin secretion in greater depth, isolated islets from control and *Gnas*^βcell–/–^ mice were studied during perifusion. Compared with controls, *Gnas*^βcell–/–^ islets had profound deficits in the insulin response to glucose, acetylcholine, and KCl (Figure 3A); it is worth noting that the insulin responses to a muscarinic receptor agonist that couples primarily through Gαq (acetylcholine) was reduced by approximately 80%, and the response to ionic depolarization (KCl) was 55% lower in *Gnas*^βcell–/–^ islets. To rectify these findings with the *Gnas*^βcell–/–^ phenotype, we measured cAMP levels in response to either acetylcholine or KCl. Surprisingly, both stimuli increased cAMP levels robustly in control islets, but a muted cAMP response was measured in response to both acetylcholine and KCl in *Gnas*^βcell–/–^ islets (Figure 3B). Islets were also treated with the phosphodiesterase inhibitor 3-isobutyl-1-methylxanthine (IBMX) to preserve intracellular cAMP levels. Control islets responded to IBMX with pronounced insulin and cAMP responses, which was greatly reduced in *Gnas*^βcell–/–^ islets (Figure 3, A and B). This suggests a deficit of cAMP generation in β cells that do not express Gαs, an inference supported by reduced PKA substrates detected in *Gnas*^βcell–/–^ islet extracts after IBMX treatment (Figure 3C). These data were further corroborated by direct measures of cAMP levels in response to glucose-dependent insulinotropic polypeptide (GIP), which was impaired in the *Gnas*^βcell–/–^ islets (Supplemental Figure 4A). We have previously shown that blocking α–to–β cell communication with Exendin (9-39) (Ex9) decreases IBMX-stimulated insulin secretion by reducing β cell cAMP levels (6). Here, we show that the effect of Ex9 to limit insulin secretion in response to IBMX is comparable to the defect in insulin secretion seen in *Gnas*^βcell–/–^ islets (Figure 3D and Supplemental Figure 4B). Moreover, Ex9 did not further reduce insulin secretion in response to IBMX in *Gnas*^βcell–/–^ islets (Figure 3D). Finally, the insulin secretory response to forskolin (FSK), a potent stimulus for adenylyl cyclase production of cAMP, was also impaired by β cell *Gnas* deletion (Figure 3E), suggesting a requisite role for Gαs in adenylyl cyclase activity independent of GPCR activity. However, when adenylyl cyclase activation was bypassed by providing a cell-permeable cAMP analog (Sp-8-BnT-cAMP), the robust stimulation of insulin secretion observed in control islets was absent in *Gnas*^βcell–/–^ islets (Figure 3F). Taken together, these results demonstrate the importance of Gαs/cAMP for the magnitude of insulin secretion in response to a variety of secretagogs and stimuli. This is further emphasized by the observation that insulin secretion rates during unstimulated conditions (2.7 mM glucose) were similar between groups and the insulin content of controls and *Gnas*^βcell–/–^ islets was similar (Supplemental Figure 2F).

While there was little difference in the basal phosphorylation of specific PKA targets (i.e., individual bands) between control and *Gnas*^βcell–/–^ islets, there was an appreciable difference in the magnitude of the phosphorylation in response to stimulation with IBMX (Figure 3C). To gain insight into the potential targets driving differences in insulin secretion, we assessed the proteomic and phosphorylation profiles of control and *Gnas*^βcell–/–^ islets under basal (PBS) or stimulated (IBMX) conditions. Due to the acute nature of these treatment exposures, proteomics was assessed based on genotypes, with treatment types pooled. While several targets associated with islet metabolism and insulin secretion (Gpd2 and Igf1r), second messenger signaling (Prkar2b and Prkcb), and extracellular matrix formation (Col1a2 and Col1a1) were higher in control islets (Supplemental Figure 5A), these changes were not attributable to any pathway, as determined by pathway analysis. Conversely, proteins involved in islet expansion (Bmp1, Chgb, and Igfbp5) were among those higher in *Gnas*^βcell–/–^ islets. Analysis of phosphorylation modifications by phosphoproteomics identified 4 groups of phosphosites associated with the response to IBMX compared with the vehicle-treated control condition between control and *Gnas*^βcell–/–^ islets (Supplemental Figure 5B), including phosphosites that were (a) stimulated by IBMX to the same degree in both groups (red); (b) significantly stimulated by IBMX in both groups, but to a greater extent in control islets (black); (c) significantly stimulated by IBMX only in control islets; and (d) significantly stimulated by IBMX in *Gnas*^βcell–/–^ islets. Pathway analysis using phosphorylated protein targets utilized Gene Ontology (GO**)** enrichment to identify potential pathways associated with differences between groups (Supplemental Figure 5C). There were 33 phosphosites similarly upregulated in both groups in response to IBMX (red dots) that did not reveal any meaningful pathways following GO enrichment (Supplemental Figure 5B); 159 phosphosites were upregulated to a greater extent in control samples after IBMX, which associated with pathways involved with intracellular signal transduction and protein transport; and 43 phosphosites were significantly upregulated in control but not *Gnas*^βcell–/–^ islets, which were also associated with pathways involved in protein transport. Interestingly, 64 phosphosites were significantly upregulated in *Gnas*^βcell–/–^ islets but not control islets, and these were associated with microtubule bundle formation. Motif analysis of the phosphosites increased by IBMX in the *Gnas*^βcell–/–^ islets did not show a PKA binding motif (Supplemental Figure 5D). By comparison, a similar analysis of the phosphosites significantly upregulated in control islets following IBMX treatment produced a clear RRXS binding motif, indicative of PKA binding (Supplemental Figure 5D). Finally, to infer the effects of IBMX treatment on kinase activity, we performed kinase-substrate enrichment analysis using phosphosite abundance data for WT and *Gnas*^βcell–/–^ islets (Supplemental Figure 5E). Kinases with a positive *z* score are predicted to be activated by the treatment, and vice versa for negative *z* scores. Akt1 and Prkaca (the catalytic subunit of PKA) were the only 2 kinases whose activities were predicted to be significantly affected by IBMX treatment in WT islets (using an FDR cutoff of 0.05). No kinases were significantly affected by IBMX treatment in *Gnas*^βcell–/–^ islets at this threshold. These findings show a lack of effective compensatory changes in *Gnas*^βcell–/–^ islets, further supporting the fundamental role of Gs-coupled signaling to β cell function.

The results of our proteomics and phosphoproteomics data are compatible with the defects in *Gnas*^βcell–/–^ islets to insulin secretagogs having both cAMP-dependent and -independent components and suggest broad downregulation of the machinery needed for insulin secretion. We hypothesized that restoration of cAMP signaling could rescue the response to cAMP-independent secretagogs. To test this, control and *Gnas*^βcell–/–^ islets were incubated in Sp-8-BnT-cAMPS for 4 days in culture, followed by a washout period of 24 hours and a perifusion experiment. *Gnas*^βcell–/–^ islets exposed to control conditions continued to show diminished rates of insulin secretion in response to high glucose, acetylcholine, KCl, and forskolin (Supplemental Figure 6). Treatment of control islets with Sp-8-BnT-cAMPS for 4 days dampened the insulin secretion response, but this exposure had no effect on *Gnas*^βcell–/–^ islets (Supplemental Figure 6). Thus, under these conditions — ex vivo treatment for a relatively short period of time — provision of exogenous cAMP did not restore the defective insulin secretion response in *Gnas*^βcell–/–^ islets.

**Figure 4:**
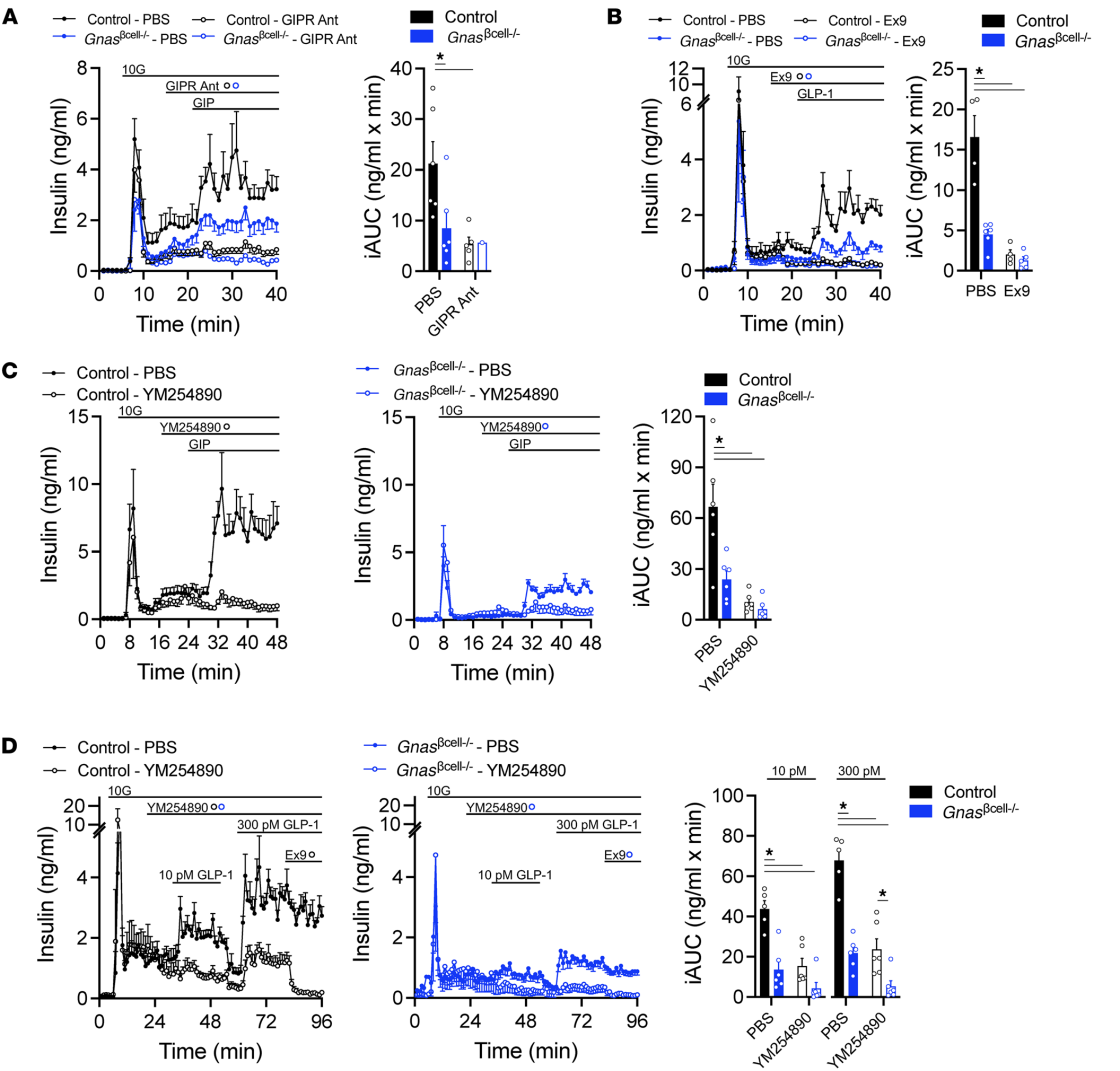
Loss of *Gnas* in β cells partially impairs incretin-stimulated insulin secretion. (**A**) Insulin secretion in response to GIP (3 nM) stimulation in control or *Gnas*^βcell–/–^ islets in the presence or absence of GIPR antibody (300 nM) and iAUC of GIP-stimulated insulin secretion (*n* = 1–6). (**B**) Insulin secretion in response to GLP-1 (10 pM) stimulation in control or *Gnas*^βcell–/–^ islets in the presence or absence of Ex9 (1 μM) and iAUC of GLP-1–stimulated insulin secretion (*n* = 5–7). (**C**) Insulin secretion in response to GIP (3 nM) stimulation in control or *Gnas*^βcell–/–^ islets in the presence or absence of the Gq inhibitor YM254890 (100 nM) and iAUC of GIP-stimulated insulin secretion (*n* = 5–6). (**D**) Insulin secretion in response to GLP-1 (10 and 300 pM) stimulation in control or *Gnas*^βcell–/–^ islets in the presence or absence of the Gq inhibitor YM254890 (100 nM) or Ex9 (1 μM) and iAUC of GLP-1–stimulated insulin secretion (*n* = 5–6). Data are shown as mean ± SEM, *P < 0.05 as indicated. Data were analyzed by 2-way ANOVA of iAUC.

To determine the importance of the Gαs/cAMP pathway for incretin receptor signaling in β-cells, control and *Gnas*^βcell–/–^ islets were perifused with GIP, with and without a GIPR antagonist (27) to establish a baseline. In control islets, GIP stimulated insulin secretion, and this was prevented by pretreatment with the GIPR antagonist (Figure 4A). In *Gnas*^βcell–/–^ islets, GIP-stimulated insulin secretion was reduced to approximately 50% of the control response and was nearly abolished by the GIPR antagonist (Figure 4A). A similar protocol was repeated with GLP-1, also using the GLP-1R antagonist Ex9. Relative to control islets, *Gnas*^βcell–/–^ islets had an approximately 70% reduction in GLP-1–stimulated insulin secretion (Figure 4B), and Ex9 completely blocked GLP-1–stimulated insulin secretion in both control and *Gnas*^βcell–/–^ islets (Figure 4B). Similar to GLP-1, the insulin response to glucagon was reduced by approximately 50% in *Gnas*^βcell–/–^ islets relative to control, and the combination of Ex9 and a glucagon receptor antagonist completely blocked insulin secretion from both control and *Gnas*^βcell–/–^ islets (Supplemental Figure 7A). Interestingly, the dual incretin receptor agonist tirzepatide had the greatest reduction (~80%) in insulin secretion in the *Gnas*^βcell–/–^ islets relative to control, an effect that was only modestly affected by Ex9 (Supplemental Figure 7B). Thus, the effects of both GIPR and GLP-1R agonists to stimulate insulin secretion were significantly reduced, but not absent, in the *Gnas*^βcell–/–^ islets, suggesting some involvement of Gαs/cAMP-independent pathways.

To determine whether residual insulin secretion in the *Gnas*^βcell–/–^ islets was due to Gαq signaling, as proposed previously by other investigators (20, 21), a selective inhibitor of Gαq-mediated signaling (YM254890) (28) was used to block incretin stimulation. In an initial experiment, YM2354890 did not directly impair the ability of incretin receptor agonists to stimulate cAMP in control islets (Supplemental Figure 7, C and D). However, treatment with YM254890 almost abolished GIP-stimulated insulin secretion in both control and *Gnas*^βcell–/–^ islets (Figure 4C). This suggests that Gαq is a key component of GIPR signaling in β cells and likely contributes to the residual activity of GIP in *Gnas*^βcell–/–^ islets. An important caveat of this interpretation is that Gαq inhibition with YM254890 is not β cell specific and likely impacts GIPR signaling in other islet cells that can regulate insulin secretion (8, 29–31). A similar approach was used to determine the contribution of Gαq to GLP-1R–stimulated insulin secretion. In these experiments, 2 different concentrations of GLP-1, 10 and 300 pM, were used since it has been reported previously that 10 pM GLP-1 stimulates insulin secretion through a Gαq/PLC/Ca^2+^ pathway, while 300 pM GLP-1 engages Gαs/cAMP/PKA (20). In control islets, blocking Gαq signaling reduced insulin secretion at both concentrations of GLP-1, but not to the degree seen with Ex9 treatment (Figure 4D). Interestingly, 10 pM GLP-1 failed to significantly stimulate insulin secretion in the presence of YM254890, while there remained noticeable increases in the response to 300 pM GLP-1. In *Gnas*^βcell–/–^ islets, inhibition of Gαq further reduced insulin secretion at both 10 and 300 pM concentrations (Figure 4D). Moreover, in the absence of β cell Gαs, Ex9 did not reduce insulin secretion further. Thus, similar to GIPR agonism, residual insulin secretion in response to GLP-1R agonism in *Gnas*^βcell–/–^ islets can be attributed to Gαq signaling.

**Figure 5:**
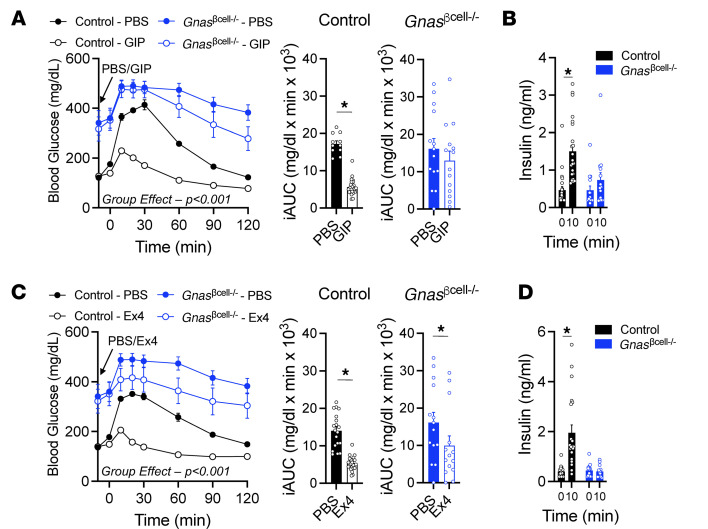
β Cell *Gnas* expression is necessary for incretin-stimulated insulin secretion in vivo. (**A**–**D**) Control (*n* = 34) or *Gnas*^βcell–/–^ (*n* = 23) mice were treated with PBS, D-Ala2-GIP (**A** and **B**), or Ex4 (**C** and **D**) at *t* = –10 minutes. Mice were then challenged with i.p. glucose (1.5 g/kg) and iAUC presented from *t* = 0. Insulin secretion in D-Ala2-GIP–challenged (**B**, *n* = 23,14) and Ex4-challenged (**D**, *n* = 21,13) mice are shown at baseline (*t* = 0) and 10 minutes after glucose challenge (*t* = 10). Data are shown as mean ± SEM, *P < 0.05 as indicated. Data were analyzed by 2-way ANOVA of glycemic curves and insulin levels or 2-tailed Student’s *t* test of the iAUCs.

To contextualize the implications of these islet experiments in vivo, we determined the effects of GIP and GLP-1 on glucose tolerance in *Gnas*^βcell–/–^ and control mice. Exogenous GIP given prior to an intraperitoneal glucose tolerance test robustly stimulated insulin secretion and decreased glycemia in control mice but did not affect either parameter in *Gnas*^βcell–/–^ mice (Figure 5, A and B). In contrast, the GLP-1R agonist Exendin-4 (Ex4) stimulated insulin secretion and lowered glycemia in control mice (Figure 5C), while in *Gnas*^βcell–/–^ mice, the effect of Ex4 on glycemia was muted but significant (Figure 5C). Interestingly, the effect of Ex4 on glucose in *Gnas*^βcell–/–^ mice occurred in the absence of a significant rise in insulin secretion (Figure 5D). This result mirrors those seen in mice with β cell deletion of the *Glp1r*, where GLP-1R agonists lower glycemia through β cell–independent mechanisms and without an increase in insulin secretion (14). From these in vivo results, it appears that the residual incretin-stimulated insulin secretion mediated by Gαq in isolated *Gnas*^βcell–/–^ islets is insufficient to have a significant impact on glycemia.

**Figure 6:**
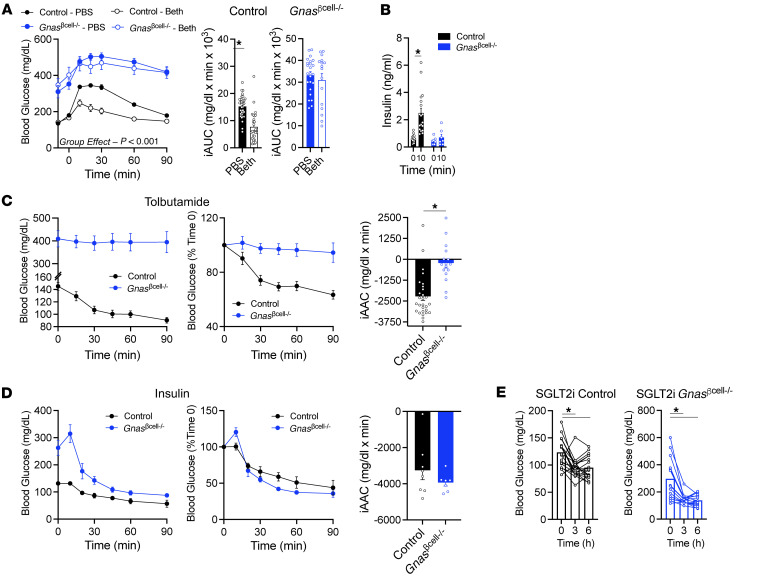
β Cell *Gnas* is necessary for glucose lowering in response to Gαs-independent, islet-targeted therapies. (**A**) Glycemia and iAUC from control (*n* = 28–34) and *Gnas*^βcell–/–^ mice (*n* = 16–23) treated with PBS or bethanechol (Beth; 2 mg/kg) by i.p. injection 10 minutes before (*t* = –10) i.p. glucose challenge (*t* = 0; 1.5 g/kg). (**B**) Insulin at baseline (*t* = 0) and 10 minutes after glucose injection (*t* = 10) in control (*n* = 18) and *Gnas*^βcell–/–^ mice (*n* = 7). (**C**) Glycemia, percent of baseline glycemia, and integrated area above the curve (iAAC) from tolbutamide (100 mg/kg) gavage in control (*n* = 28) and *Gnas*^βcell–/–^ (*n* = 16) mice. (**D**) Glycemia, percent of baseline glycemia, and iAAC from insulin tolerance test (1 U/kg) in control (*n* = 8) and *Gnas*^βcell–/–^ (*n* = 7) mice. (**E**) Glycemia after dapagliflozin (10 mg/kg) gavage in control (*n* = 19) and *Gnas*^βcell–/–^ (*n* = 14) mice. SGLT2i, SGLT2 inhibitor. Data are shown as mean ± SEM, *P < 0.05 as indicated. Data were analyzed by 1-way ANOVA of glycemia (**E**), 2-way ANOVA of glycemic curves and insulin levels, or 2-tailed Student’s *t* test of the iAUCs.

Finally, we wanted to determine whether the hyperglycemia present in *Gnas*^βcell–/–^ mice was responsive to treatments that (a) target β cells through non-Gαs pathways or (b) are independent of β cells. In response to bethanechol, a muscarinic receptor agonist that signals through Gαq, there was robust stimulation of insulin secretion with consequent reductions in blood glucose in control mice, but no effect in *Gnas*^βcell–/–^ mice (Figure 6, A and B). Similar results were noted with tolbutamide, a sulfonylurea that closes K_ATP_ channels to initiate β cell depolarization and insulin secretion (Figure 6C). Finally, islet-independent mechanisms were tested, including exogenous insulin (Figure 6D) and the SGLT2 inhibitor dapagliflozin (Figure 6E). Both interventions robustly lowered glycemia in control and *Gnas*^βcell–/–^ mice. These results emphasize that β cell function in *Gnas*^βcell–/–^ mice is severely impaired, and all interventions that require β cell activity fail to lower glycemia.

## Discussion

Incretin receptor agonists have been a significant advance in the treatment of diabetes, obesity, and comorbidities (15). As the understanding of incretin biology has progressed, it has become evident that the intracellular signaling mechanisms of their receptors are far more complex than simply the well-established Gαs/cAMP pathway. For example, one differentiating signaling node that has separated semaglutide from tirzepatide activity at the GLP-1R is the recruitment of β-arrestin proteins, which translates to functional differences in intracellular signaling, receptor internalization, and potentially additional cellular outcomes (32). Expanding on this are reports that GLP-1R can signal in β cells to control insulin secretion and glucose homeostasis through Gαs/cAMP-independent mechanisms (20, 21). The discovery of biased signaling at GPCRs has enabled the engineering of peptides that can preferentially engage specific downstream signaling nodes emanating from an individual receptor (33). Given the diminished effect of native incretins in T2D (34), which primarily engage the Gαs/cAMP pathway, it seems reasonable to propose that preferential engagement of alternative pathways would be an effective strategy to leverage incretin receptor activity to treat people with diabetes. Notably, recent studies indicate that coupling of GPCRs to multiple G protein subfamilies is much more common than previously thought (35). As such, it is first important to clearly delineate the contribution of Gαs in incretin receptor activity. Here, we describe how Gαs activity in β cells is essential for maintaining cAMP levels, glucose-stimulated insulin secretion, the response to incretin receptor agonists, and even the insulin response to insulin secretagogs that work independently of Gαs. Moreover, our data illustrate that while Gαs-independent pathways contribute to insulin secretion, this action is insufficient to correct hyperglycemia or mediate the full response to incretin receptor agonists. Collectively, these data emphasize that the capacity of β cells to increase Gαs/cAMP is fundamental for normal function and metabolic regulation. Beyond physiology, this property of incretin signaling should remain a central consideration in the development of incretin-based drugs.

Loss-of-function mutations in *GNAS* produce resistance to multiple hormones, resulting in several distinct clinical syndromes, such as pseudohypoparathyroidism 1A, which includes several metabolic features (36–39). However, understanding of this condition exemplifies that while *GNAS* is ubiquitous in its expression, its regulation of several gene products and variable parental imprinting in different tissues (40) blur attribution of specific phenotypic characteristics to lack of Gαs activity in any one cell type in humans (40). In this context, it is interesting to note that analysis of *GNAS* expression across a cross-sectional sample of human islets revealed that (a) *GNAS* expression positively correlated with insulin secretion, and (b) *GNAS* levels were lower in islets from patients with diabetes compared with those from nondiabetics (41). Additional studies used siRNA to silence *Gnas* expression in rat INS-1 β cells, producing reductions in insulin secretion in response to high glucose and forskolin (41) and thus mirroring the results of our studies. Taken together, these data suggest that *GNAS* mutations or variants that reduce Gαs activity could contribute to metabolic disorders, including suboptimal insulin secretion.

Our studies phenocopy aspects of previous models with Gαs deletion in β cells (22–24), most notably the profound hyperglycemia. However, a distinguishing feature of our work is the temporal deletion of the *Gnas* gene in adult mice that does not lead to significant changes in β cell development or islet morphology. Thus, our model is cell specific and emphasizes functional aspects of β cell secretion. It is important to note that the *MIP-Cre^ERT^* model we utilized to achieve temporal deletion of *Gnas* contains the human growth hormone mini gene, which has been shown to positively impact β cell function in the setting of extreme stress (42). We found increased rates of insulin secretion in *MIP-Cre^ERT^* islets compared with islets from WT littermate controls (Supplemental Figure 1A). However, this increase in β cell function did not lead to improvements in glucose tolerance during physiological or pharmacological interventions in chow-fed mice (Supplemental Figure 1, B–D). This observation agrees with previously published results (42, 43), suggesting that the *MIP-Cre^ERT^* transgene has effects beyond tissue-specific generation of recombinase that need to be understood relative to study outcomes. For our study, the relevant effect is a modest elevation in β cell function during islet perifusion (44). However, given both the magnitude and directionality of the impaired β cell function seen in *Gnas*^βcell–/–^ mice, it is unlikely that this potential confounder from the *MIP-Cre^ERT^* model affects the interpretations of our results. The *Gnas*^βcell–/–^ mice described here have low and unchanging levels of circulating insulin and develop severe hyperglycemia. However, they do not demonstrate the characteristics of other insulinopenic mouse models that typically have weight loss, lean tissue wasting, and often premature death. In fact, the *Gnas*^βcell–/–^ mice had equivalent weight gain to control mice on a HFD, suggesting that their insulin deficit was not so severe as to prevent tissue anabolism. This indicates that a more nuanced interpretation is required when evaluating the hypothesis that relative hypoinsulinemia is a protective mechanism against diet-induced obesity (45). Indeed, other than hyperglycemia, the *Gnas*^βcell–/–^ mice thrived, with modest hyperphagia compensating for renal glucose loss. The phenotype of this mouse is actually most akin to patients with maturity-onset diabetes of the young type 2, who display hyperglycemia from birth but without additional complications and often do not require medical intervention (46).

GLP-1R and GIPR have long been known to couple with Gαs to exert their effects on insulin mobilization and secretion. There is also evidence that GLP-1R couples to Gαq signaling to stimulate insulin secretion, particularly in physiological ranges of GLP-1R agonism (20). More recently, the idea of a Gαs-Gαq switch has been proposed, describing how the GLP-1R adapts to chronic hyperglycemia by preferentially coupling to Gαq to support insulin secretion. These studies also reported that the GIPR did not possess this flexibility in signaling, potentially explaining the decrease in GIPR activity and the incretin effect in T2D (21). Our current study corroborates aspects of this work. First, we show that low (10 pM) concentrations of GLP-1 rely to a greater extent on Gαq signaling than higher concentrations of GLP-1 (300 pM) (Figure 4, B and D). However, even at low concentrations of GLP-1, Gαs still accounts for the majority of insulin secretion. Second, there is residual insulin secretion in response to both GLP-1 and GIP in *Gnas*^βcell–/–^ mice, which display a level of chronic hyperglycemia that is similar to previous reports (21). We document in isolated islets that this residual insulin secretion can be attributed to Gαq signaling. However, in vivo, this residual insulin secretion is insufficient to regulate glucose levels in response to incretin peptides (Figure 5). Thus, the results of our in vivo experiments are in keeping with findings previously reported in β cell knockout models of *Gipr* (47) or *Glp1r* (14), emphasizing the limited or absent incretin signaling in β cells of *Gnas*^βcell–/–^ mice.

Deletion of β cell Gαs signaling greatly reduced the activity of both incretin receptors to a comparable level. In isolated islets, the decrease in insulin secretion in response to either GIP or GLP-1 was similar and appeared to be equally sensitive to Gαq inhibition. Interestingly, the deficient incretin receptor signaling in *Gnas*^βcell–/–^ islets also translated to similar impairments of insulin secretion in vivo in response to both GIPR or GLP-1R agonism. However, GLP-1R agonism retained some ability to reduce glycemia in vivo, whereas GIPR agonism did not. This aligns with our previous reports showing that GLP-1R agonists have a component of glucose-lowering that is independent of β cell activity (14), while GIPR agonists do not (47). Collectively, these results emphasize that the magnitude and directionality of incretin receptor activity is equally impacted by deletion of Gαs in β cells but that differential mechanisms between incretin receptors to lower glycemia in vivo remain intact.

Finally, an unexpected outcome of these studies was the loss of insulin secretion in response to agents that are typically thought of as independent of GαS activity. This includes impaired insulin secretion in response to muscarinic receptor agonists (acetylcholine and bethanechol) (Figure 3A and Figure 6A), depolarization with K_ATP_ closure (KCl and sulphonylurea) (Figure 3A and Figure 6C), and directly generating cAMP with forskolin (Figure 3E) or treatment with a cell-permeable cAMP analog (Figure 3F). These observations can be partially explained by the effects of some of these stimuli (acetylcholine and KCl) to produce a rise in cAMP in control islets, which is muted in *Gnas*^βcell–/–^ islets (Figure 3B). However, it is also interesting to note that the robust insulin secretion in control islets obtained with direct activation of adenylyl cyclase with forskolin or the addition of a cAMP analog was severely muted in *Gnas*^βcell–/–^ islets (Figure 3, E and F). These results point to a defect beyond the generation of cAMP arising from loss of Gαs signaling. Indeed, our proteomics results revealed several phosphosites on proteins involved in calcium signaling (Nucb2, Sptan1, and Crebbp) and membrane trafficking (Map2, Vamp4, and Sptan1) that were upregulated in *Gnas*^βcell–/–^ islets but not control islets, suggesting mechanisms were engaged to compensate for inefficient secretory capability. This hypothesis aligns with the observation that the insulin granules were larger in *Gnas*^βcell–/–^ islets (Figure 1F), a feature previously described for defects in secretion and docking of the readily releasable pool of insulin-containing vesicles (48). Related to this, Camk2a was predicted to be the kinase most highly induced by IBMX in *Gnas*^βcell–/–^ islets, with a *z* score that was slightly higher than in WT islets. This could reflect a compensatory increase in Gαq activity in *Gnas*^βcell–/–^ islets to signaling through calcium. *Gnas*^βcell–/–^ islets also displayed a significant increase in Prkaca peptide abundance compared with WT controls, suggestive of another means of compensation for reduced Gαs abundance/activity. However, any compensation via these mechanisms was unable to meaningfully restore insulin secretion in the absence of Gαs.

In summary, the results presented here emphasize the importance of Gαs signaling through cAMP as having more than simply a modulatory function on insulin secretion, but it is also essential for the stimulated responses that control hyperglycemia. We show that complete loss of Gαs in postdevelopment β cells impairs insulin secretion in response to any islet-directed stimulus, independent of changes in islet number, insulin-positive area, or basal insulin levels. This led to profound, persistent hyperglycemia, particularly in male mice. Notably, Gαq signaling was unable to compensate for loss of Gαs signaling, further demonstrating the critical role of cAMP to islet function and overall glucose homeostasis. Together, these results shine a light on the Gαs/cAMP pathway as the key regulator of β cell function and support continued pursuit of therapeutic agents that target this pathway.

## Methods

### Sex as a biological variable.

Both male and female mice were used for these studies. The results were similar, and the results for the male mice are shown. For islet function studies, islets from both male and female mice were used. No differences between sex were found, and the results were pooled.

### Reagents.

IBMX, FSK, acetylcholine, carbamyl-β-methylcholine chloride (bethanechol), and tolbutamide were purchased from Sigma. Ex9 was synthesized and purchased from GenScript. Mouse GIP and D-Ala2-GIP were purchased from Phoenix Pharmaceuticals. GLP-1 was purchased from Bachem. GIPR antagonist was provided by Eli Lilly. Gq/11 signaling inhibitor YM-254890 was purchased from Tocris Bioscience. Ex4 was purchased from MedChemExpress. Tirzepatide was provided by Eli Lilly. Dapagliflozin was purchased from Advanced ChemBlocks Inc.

### Animals.

Experiments were performed in 8- to 28-week-old mice on a C57Bl6/J background. Mice were housed under a 12-hour light/dark cycle and provided free access to a normal chow diet. Mice carrying LoxP sites on exon 1 of *Gnas* (guanine nucleotide binding protein, α stimulating) (*Gnas*^fl/fl^) (24, 49) were crossed with MIP-Cre^ERT^ (*MIP-Cre*) mice to generate β cell–specific deletion of Gnas (*Gnas*^βcell–/–^). Controls were a combination of WT, MIP-Cre^ERT^ (Cre allele only), and *Gnas*^fl/fl^ (floxed allele only). WT and MIP-Cre^ERT^ mice were littermates, while *Gnas*^fl/fl^ and *Gnas*^βcell–/–^ were littermates. Because these 3 lines had identical glucose tolerance, they were pooled for in vivo experiments. For perifusion experiments, controls were *Gnas*^fl/fl^ littermates. At either 6–8 or 20–22 weeks of age, all mice received oral tamoxifen treatment for 4 consecutive days (5 mg/day). Tamoxifen was dissolved in corn oil at 50 mg/mL. Control groups produced similar experimental results and were therefore grouped or used interchangeably. Our study examined male and female animals, and similar findings are reported for both sexes. All animals were maintained and used in accordance with protocols approved by the Duke University IACUC.

### Histology.

Pancreas was collected from mice at 6 weeks after tamoxifen treatment. Briefly, pancreas was fixed in 10% formalin overnight at 4°C, before transferring to 70% ethanol until paraffin embedding. Sections were cut at 5 μm thickness at 2 different depths through the pancreas, and serial sections were stained for insulin (Cell Signaling; 3014S) or Chromogranin A (Thermo Fisher Scientific; PA5-32349). Antibody-positive areas and total pancreas area were measured using QuPath software (50) by a blinded reviewer. Each data point reported is the average of the 2 images taken from a single pancreas.

### Direct stochastic optical reconstruction microscopy of insulin granules.

Control and *Gnas*^βcell–/–^ islets were labeled with rabbit anti-insulin (Cell Signaling; 3014S; RRID:AB_2126503) overnight at 4°C. Following PBS washes, islets were incubated with goat anti-rabbit Alexa Fluor 647 (Thermo Fisher Scientific; A-21245; RRID:AB_2535813) for 2 hours at room temperature. Labeled islets were then placed on a cavity slide, submerged in STORM buffer (Abbelight), and sealed using a 170 μm coverslip + EcoSil dental resin (Picodent; catalog 1300 6100). Samples were imaged at approximately 30 nm lateral resolution using TIRF mode on an Evident/Abbelight SAFe 180 system, and a ×100, 1.5 NA Olympus UPLAPO100XOHR objective. Alexa Fluor 647 was pumped to the dark state using an Oxxius laser combiner and 500 mW, 640 nm diode laser, before initiation of photoblinking. A 405 nm laser was slowly ramped up during the acquisition to increase transition back to the dark state. Single-molecule events were recorded using an LP650 filter with an integration time of 50 ms on a Hamamatsu ORCA-Fusion sCMOS camera for 50,000 frames. Localizations were extracted and images reconstructed using Abbelight NEO software v39. Density-Based Spatial Clustering of Applications with Noise was used to determine localization clustering, implemented in Abbelight NEO software v39, with ε = 25 nm (the average precision of the data) and minPts = 8.

### Structured illumination microscopy of insulin granules.

Paraffin-embedded pancreas sections from control and *Gnas*^β-cell–/–^ mice were deparaffinized and rehydrated through a series of alcohol washes followed by blocking with PBS with 2% BSA for 1 hour at room temperature. Sections were incubated with rabbit anti-insulin (Cell Signaling; 3014S) overnight at 4°C. After PBS washes, sections were incubated with anti-rabbit Alexa 488 (Thermo Fisher Scientific; A-21206) for 2 hours at room temperature. Sections were then mounted in Vectashield Hardset mounting medium with DAPI. Images were acquired at approximately 100 nm lateral resolution using a Nikon N-SIM S microscope SR, HP Apo TIRF ×100 1.49 NA/oil immersion objective, and ORCA-Flash 4.0 sCMOS camera with online deconvolution. Excitation was delivered at λ = 405 and λ = 488, and emitted signals were detected at λ = 400–450 and λ= 500–550 for DAPI and Alexa Flour 488, respectively. Insulin granule size and area were quantified using ImageJ (NIH). Briefly, a region of interest (ROI) was drawn to acquire the area measurement around each granule. For percent area occupied, a ROI was drawn around individual β cells to acquire total insulin granule area/total cell area.

### Islet isolation.

Islets were isolated from mice using previously published methods (51). Briefly, the pancreas was inflated by injecting 0.8 mg/mL collagenase V (Sigma) in HBSS through the pancreatic duct. The inflated pancreas was then removed and digested in collagenase V for 12 minutes at 37°C, with gentle agitation every 4 minutes. Digestion was quenched with cold RPMI (2 mM l-glutamine, 11.1 mM glucose, and 0.25% BSA), and islets were separated after passing digested tissue through a filter and using a Histopaque gradient (Sigma). Islets were allowed to recover overnight in RPMI with 10% FBS prior to all experiments.

### Islet dispersion and islet cell enrichment.

After overnight recovery, islets from each mouse were washed in PBS and incubated in Accutase (Sigma) for 12–15 minutes at 37°C with intermittent vortexing, and digestion was quenched with cold RPMI. Islet cells were then centrifuged for 3 minutes, 350*g*, at 4°C. RPMI was then aspirated and islets washed with sorting buffer (RPMI 1640 without phenol red, 11.1 mM glucose, 1% FBS, 1% penicillin/streptomycin, 2 μM HEPES, and 10 units/mL DNase). Islets were washed again in sorting buffer before FACS. Dispersed islets were filtered through 30 μM mesh and FACS using a Beckman-Coulter MoFlo Astrios or analyzed using an Attune NxT Analyzer (Thermo Fisher Scientific). Forward and side scatter were used to separate single cells from debris and doublets. For FACS, live islet cells were separated by autofluorescence and side scatter into α, β, and δ cell populations (52, 53).

### RNA extraction and reverse transcription PCR.

Sorted islet cells were collected into TRIzol for RNA extraction, and 100 ng of RNA was used to synthesize DNA. Quantitative PCR was run using Taqman reagents and primers, and data were analyzed by calculating ΔΔCT and normalized to *Ppia*. Data are shown as fold change relative to whole islet lysates in control islets.

### Islet perifusion.

After isolation and overnight incubation, islets were handpicked in equal numbers (75–100 islets) and placed into perifusion chambers (BioRep) containing 2.7 mM glucose in KRPH buffer (140 mM NaCl, 4.7 mM KCl, 1.5 mM CaCl_2_, 1 mM NaH_2_PO_4_, 1 mM MgSO_4_, 2 mM NaHCO_3_, 5 mM HEPES, and 1% fatty acid–free BSA; pH 7.4) with 100 μL Bio-Gel P-4 Media (Bio-Rad). Islets were perifused in the BioRep Per-04 or Per-05 system and equilibrated in 1% BSA–containing 2.7 mM glucose KRPH buffer for 24 minutes, followed by 24 minutes equilibration in 0.1% BSA–containing 2.7 mM glucose KRPH buffer. Islets were then perifused in 0.1% BSA–containing KRPH buffer at dosing and intervals defined in each figure.

### Islet cAMP and Ca^2+^ imaging.

*Gnas*^βcell–/–^ and control islets were transduced with adenoviruses encoding for CAMPER-SH187 (54) and jRGECO1a (55). Ucn3-Cre (MMRRC:037417) × Rosa26-lsl-CAMPER (JAX:032205) islets (56–58) were transduced with the adenovirus encoding for jRGECO1a. As previously described (59, 60), islets were seeded in custom-made polydimethylsiloxane perfusion chambers bonded to 35 mm glass-bottomed dishes (Mattek; P35G-1.5-14-C) and allowed to recover overnight. Islets were continuously perfused with Krebs-Ringer buffer and treatments as indicated, and their cAMP and calcium responses were monitored over time using a Nikon Eclipse Ti2 microscope at ×60 magnification. For cAMP imaging, the islets were excited with a 445 nm laser line while simultaneously detecting CFP and YFP with 2 parallel detectors. For calcium imaging, the islets were excited with a 561 nm laser line. Forskolin and KCl were included at the end of each imaging trace as a positive control of cell viability and the ability to elicit a cAMP and calcium response, respectively. ROIs were drawn around cells, and the fluorescence intensity in each ROI was measured in NIS-Elements (Nikon) to determine cAMP and calcium activity.

### In vivo tolerance tests and pharmacologic interventions.

The i.p. glucose, insulin, and meal tolerance tests were performed after a 5-hour fast. Glucose was administered at 1.5 g/kg in PBS, insulin (Humalog) at 1 U/kg in PBS, and vanilla liquid Ensure at 10 μL/g. D-Ala2-GIP was administered i.p. at 4 nmol/kg in PBS (6; 47), Ex4 was administered i.p. at 1 nmol/kg in PBS, and bethanechol was administered i.p. at 2 mg/kg in PBS. All were administered 10 minutes before glucose injection. Tolbutamide was delivered by oral gavage at 100 mg/kg. Mice were fasted for 3 hours prior to dapagliflozin gavage (10 mg/kg) and remained fasted throughout the experiment. For fast-refeed experiments, mice were fasted overnight for approximately 16 hours, and blood was collected at baseline and 60 minutes after introduction of chow diet to the cage. Blood glucose was measured using a glucometer (Contour). EDTA-coated capillary tubes were used for collection of blood. Insulin and proinsulin were measured from plasma by ELISA (Mercodia).

### Western blot analysis.

All islets from 4 mice were pooled for each sample. Islets were placed into 1.5 mL tubes and treated with 2.7 mM glucose in KRPH buffer containing 0.1% BSA with 100 μM IBMX or 0.1% DMSO (control) for 5 minutes. After incubation in treatment, buffer was quickly removed, and islets were flash frozen in liquid nitrogen and stored at –80°C until use. Islets were lysed in RIPA buffer containing protease (Fisher) and phosphatase (Cell Signaling) inhibitors. Protein was quantified with a BCA assay (Thermo Fisher Scientific), and SDS-PAGE was performed. Membranes were blocked in 4% BSA in TBST and then incubated in phospho-PKA substrate (Cell Signaling; 9624). Immunoblots were developed with ECL substrate (Bio-Rad) and imaged in a ChemiDoc imager (Bio-Rad).

### Preparation of TMT-labeled samples.

The remaining lysates from Western blot preparation were prepared for tandem mass tag (TMT) labeling. Approximately 75 μL of islet lysates was diluted with 25 μL of 20% (w/v) SDS in triethylammonium bicarbonate (TEAB) buffer, pH 8.5, followed by spike-in of bovine casein at 2:1 ratio in the *Gnas*^βcell–/–^ versus control. Ten microliters of 100 mM DTT was added, and samples were reduced at 80°C for 10 minutes. After cooling, samples were alkylated with 25 mM iodoacetamide in the dark for 30 minutes at room temperature. SDS was removed, and samples were digested with 1:10 of SEQUENZ modified trypsin (Worthington) using an S-trap mini device (Protifi) according to the manufacturer’s instruction. Recovered peptides were lyophilized to dryness. Lyophilized peptides were reconstituted in 50 μL of 200 mM TEAB buffer, and peptides were labeled with 10 μL of TMT16 reagents. After 2 hours, reactions were quenched with 2.5 μL of 5% hydroxylamine for 15 minutes, and all samples were mixed, split into 3 × 200 μg aliquots, and lyophilized. A separate 10 μg aliquot was lyophilized for the unenriched analysis. For phosphopeptide enrichment, 2 aliquots were reconstituted in 80% acetonitrile (MeCN)/1% trifluoroacetic acid (TFA) containing 1 M glycolic acid (buffer A), and phosphopeptides were enriched using GL Sciences p10 TiO tips following the manufacturer’s instructions. After loading, tips were washed twice with buffer A, followed by 2 times with 80% MeCN/1% TFA before elution with 20% MeCN/5% aqueous ammonia. An additional aliquot was enriched using glutamic acid as an excluder (61). Finally, peptides were desalted using C18 State Tips, lyophilized, and reconstituted in 12 μL of 10 mM citrate in 1% TFA/2% MeCN (for phosphopeptides) or 20 μL of 1% TFA/2% MeCN (unenriched).

### Quantitative mass spectrometry.

One-dimensional liquid chromatography, tandem mass spectrometry was performed on 4.5 μL of each of the phospho-enriched factions or on 1 μg of unenriched factions. Samples were analyzed using an M-Class UPLC system (Waters) coupled to an Exploris 480 high-resolution accurate mass tandem mass spectrometer (Thermo Fisher Scientific) via a nanoelectrospray ionization source and FAIMS Pro Interface. Samples were trapped on a Symmetry C18 180 μm × 20 mm trapping column (2 μL/min at 99.9/0.1 v/v H_2_O/MeCN) followed by an analytical separation using a 1.7 μm Acquity HSS T3 C18 75 μm × 250 mm column (Waters) with a 90-minute gradient of 5% to 30% MeCN with 0.1% formic acid at a flow rate of 400 nL/min and column temperature of 55°C. Data collection on TMT-labeled samples was performed in data-dependent acquisition mode with 2 FAIMS compensation voltages (e.g., –40/–60, –45/–65, –50/–70, and –30/–80) for a total of 6 injections at 120,000 resolution (at *m*/*z* 200) full MS scan from *m*/*z* 375 to 1,600 with a normalized AGC target of 300%, peptide monoisotopic peak determination, an intensity threshold of 5E3 ions, precursor fit of 70% with 0.7 *m*/*z* fit window, charge state of 2–5, and 45 s dynamic exclusion. Tandem mass spectra were acquired at 45,000 or 60,000 resolution, an isolation width of 0.7 *m*/*z*, NCE of 36, AGC target of 300%, and maximum IT of 200 ms. Unenriched data were collected with 2 compensation voltages (–40/–60 × 2, –50/–70, and –30/–80) for a total of 4 injections.

### Phosphoproteomic data analysis.

TMT data were analyzed using FragPipe 19 (https://fragpipe.nesvilab.org/) using default parameters for TMT16 or TMT16-phos (62). Database searching was performed against the UniProt database Mus musculus taxonomy (downloaded on July 6, 2022) and appended with contaminant sequences and an equal number of reverse decoys (34,460 total entries). TMT-integrator used defaults with “allow unlabeled” peptide N-terminus and median aggregation of peptide spectral match intensities for protein quantification median centering variance scaling in FragPipe was utilized for normalization. Statistical analysis used the generalized linear model function in edgeR (63, 64). Pathway analysis was performed using the Database for Annotation, Visualization, and Integrated Discovery v2025_1 (65, 66). Phosphosite motif analysis was performed on PhosphoSitePlus using identified amino acid sequences and assessment with motif and logo analysis tools. Kinase-substrate enrichment analysis (67) was performed on phosphoproteomics data using the *KSEAapp* R package (68). Kinase-substrate interaction data were downloaded from PhosphoSitePlus (https://www.phosphosite.org/homeAction.action). Raw and processed data, as well as associated metadata, have been uploaded and can be accessed at https://proteomecentral.proteomexchange.org/cgi/GetDataset?ID=PXD063902

### Statistics.

All data are expressed as mean ± SEM. Statistical analyses were performed using GraphPad Prism 10. A 2-tailed Student’s *t* test or 2-way ANOVA was performed, depending on the experimental design, with a Bonferroni’s post hoc analysis. *P* < 0.05 was determined to identify statistically significant differences.

### Study approval.

All mouse procedures were approved and performed in accordance with the Duke University IACUC.

### Data availability.

The data that support the findings of this study are available from the corresponding author upon reasonable request as well as in the Supporting Data Values file.

## Author contributions

Conceptualization was provided by MEC, DAD, and JEC. Investigation was provided by MEC, DB, AS, MYC, SMG, KV, AB, SLL, JCLT, AH, FC, ECR, and MWF. Data were analyzed by MEC, AB, MWF, MJM, MOH, DJH, DAD, and JEC. Resources were provided by JDD, LSW, MAH, FSW, KWS, DAD, and JEC. AC contributed essential experimental data to the revised version. The original draft of the manuscript was written by MEC, DAD, and JEC. Review and editing of the manuscript was done by MEC, DB, AS, MYC, SMG, KV, AB, JDD, SLL, JCLT, AH, FC, ECR, MWF, LSW, MAH, MJM, FSW, MOH, KWS, DJH, DAD, and JEC.

## Supplementary Material

Supplemental data

Unedited blot and gel images

Supporting data values

## Version 1 06/17/2025

In-Press Preview

## Version 2 08/15/2025

Electronic publication
