# Supplementary material for: β Cell Gαs signaling is critical for physiological and pharmacological enhancement of insulin secretion
Source: J Clin Invest. 2025 Jun 17;135(16):e183741. doi: 10.1172/JCI183741 (PMC12352888; doi:10.1172/JCI183741)
Supplement: Supplemental data [file jci-135-183741-s197.pdf]

Supplemental Figure 1

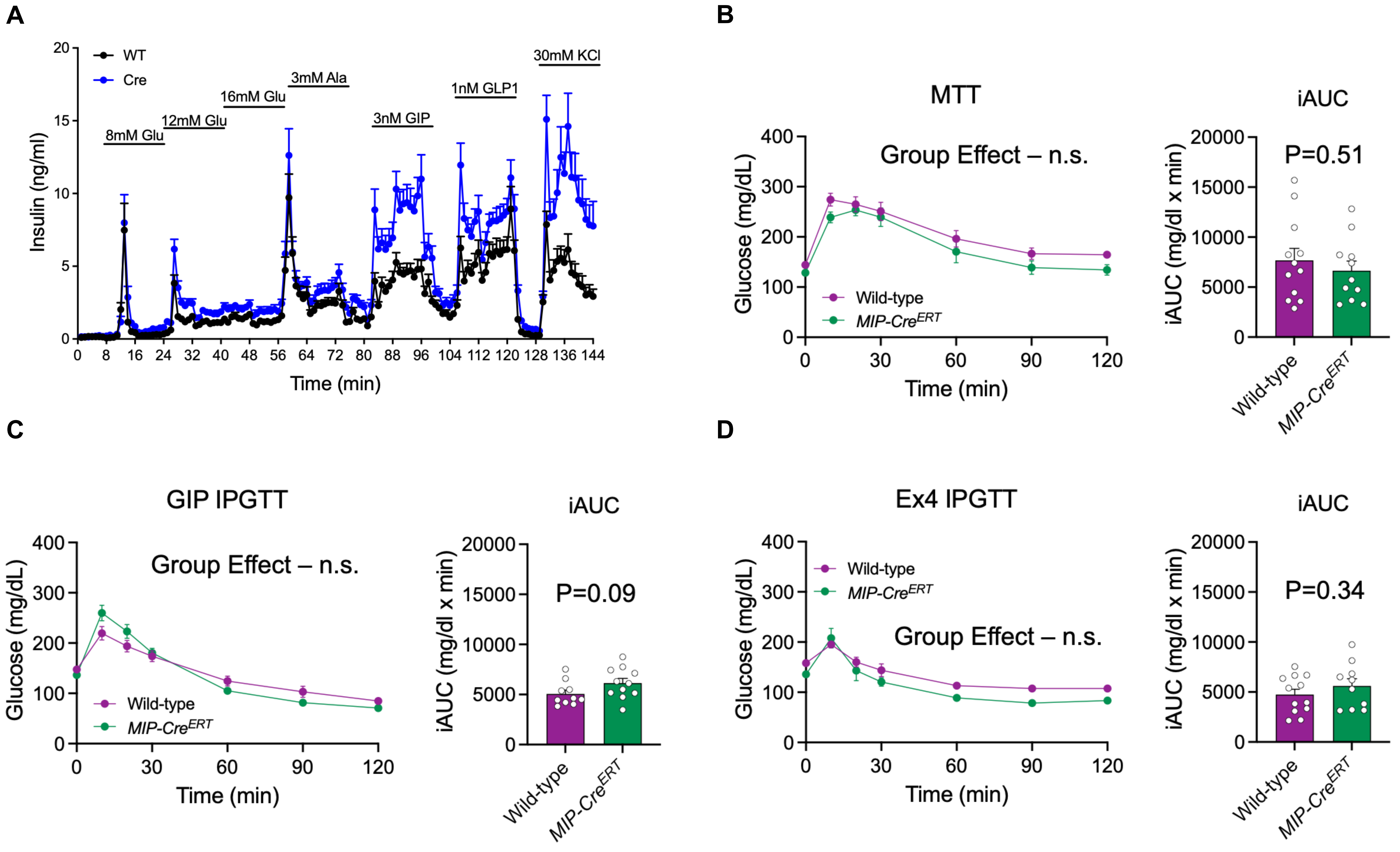

Supplemental Figure 1. Insulin secretion in islets isolated from wild-type (WT) and *MIP-Cre*^{ERT} mice. (n=6). Mixed meal tolerance test in WT and *MIP-Cre*^{ERT} mice (n=12, 11). (C) Intraperitoneal glucose tolerance test (IPGTT) in mice pretreated with 4 nmol/kg GIP. (n=10, 11). (D) IPGTT in mice pretreated with 1 nmol/kg Ex4 (n=12, 10). Data are shown as mean ± SEM, * - $p<0.05$ as indicated. Data were analyzed by student's t-test.

Supplemental Figure 2

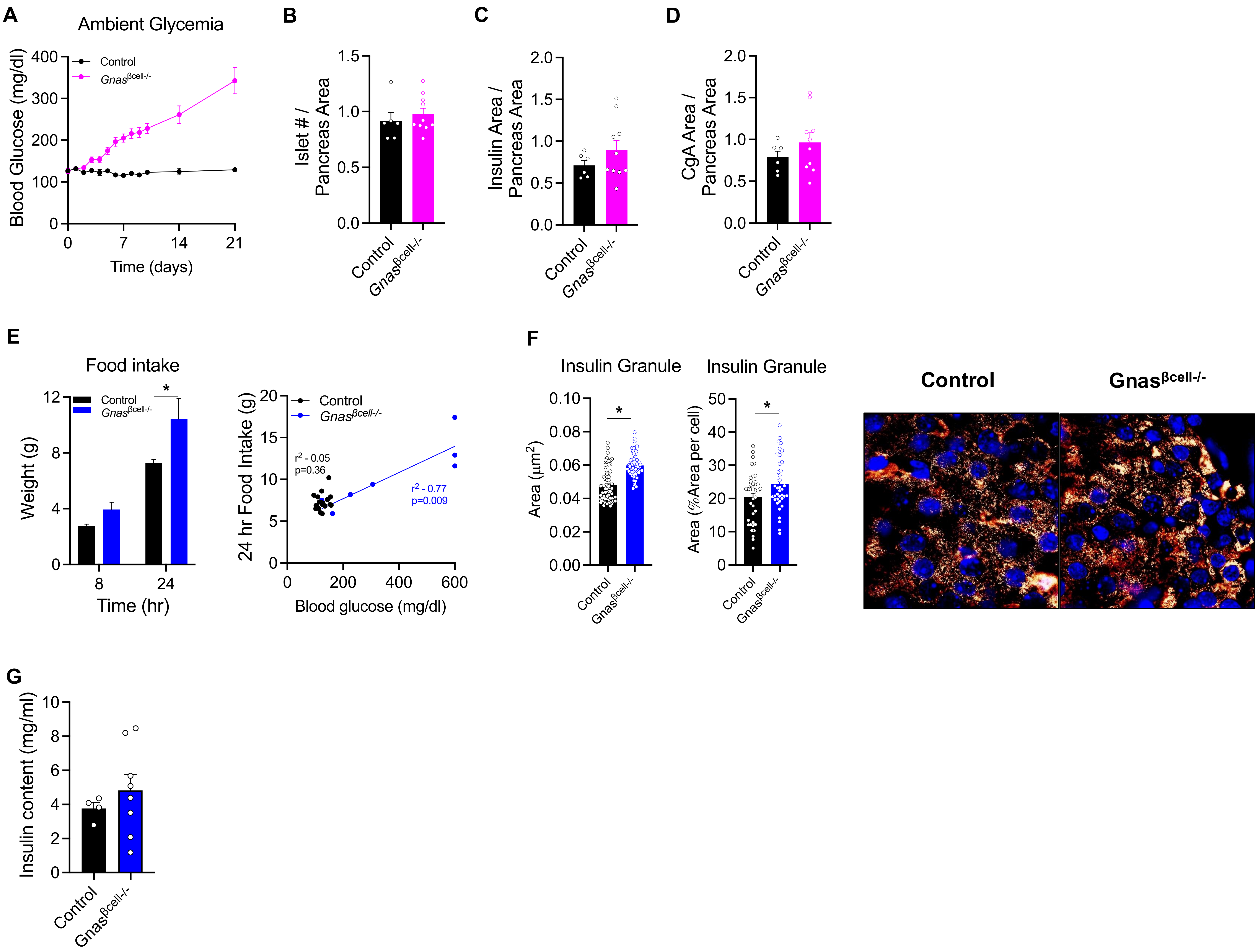

Supplemental Figure 2. (A) Blood glucose in mice given tamoxifen at 20-24 weeks of age. (n=12, 17) (B-D) Histological analysis of islet number, beta-cell area, and chromogranin A (CgA). (n=6, 10) (E) Total food consumed over an 8- or 24-hr period. (F) Insulin granule area (left) and % cell area occupied by insulin granules (middle) from control and *Gnas*$^{\beta cell-/-}$ mice, together with representative images (right) (area, n= 20 granules per cell from 45 cells from 3 mice per group; % area occupied, n = 35 cells from 3 mice per group). (G) Insulin content from isolated islets (n=4,8). Data are shown as mean ± SEM. Data were analyzed by 2-way ANOVA or regression analysis (A) or student's t-test (B,C). * - p<0.05 as indicated, n=18, control; n=7, *Gnas*$^{\beta cell-/-}$ mice.

Supplemental Figure 3

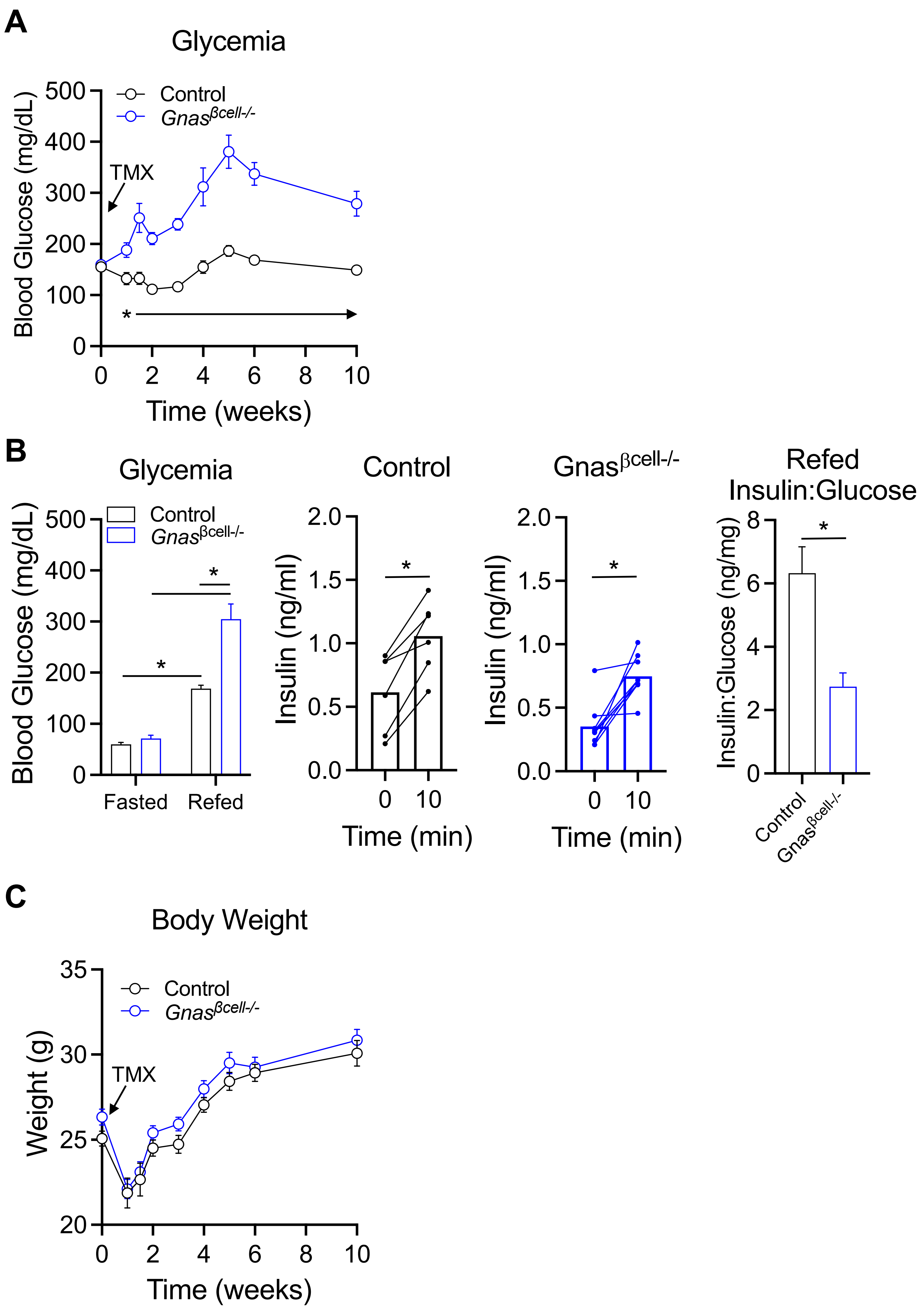

Supplemental Figure 3. Impact of high-fat diet in control and *Gnas*$^{\beta cell-/-}$ mice. (A) Ambient blood glucose over a 10-week period following tamoxifen treatment in mice fed a high-fat diet. (B) Fasted and refed glycemia, insulin and the insulin:glucose after 10 weeks of high-fat diet. (C) Body weight over the 10-week period of high-fat diet. Data are shown as mean ± SEM. Data were analyzed by 2-way ANOVA (A, B, C) or student's t-tests (B), as appropriate. * - $p<0.05$ as indicated. N=6, control; N=7, *Gnas*$^{\beta cell-/-}$ mice.

Supplemental Figure 4

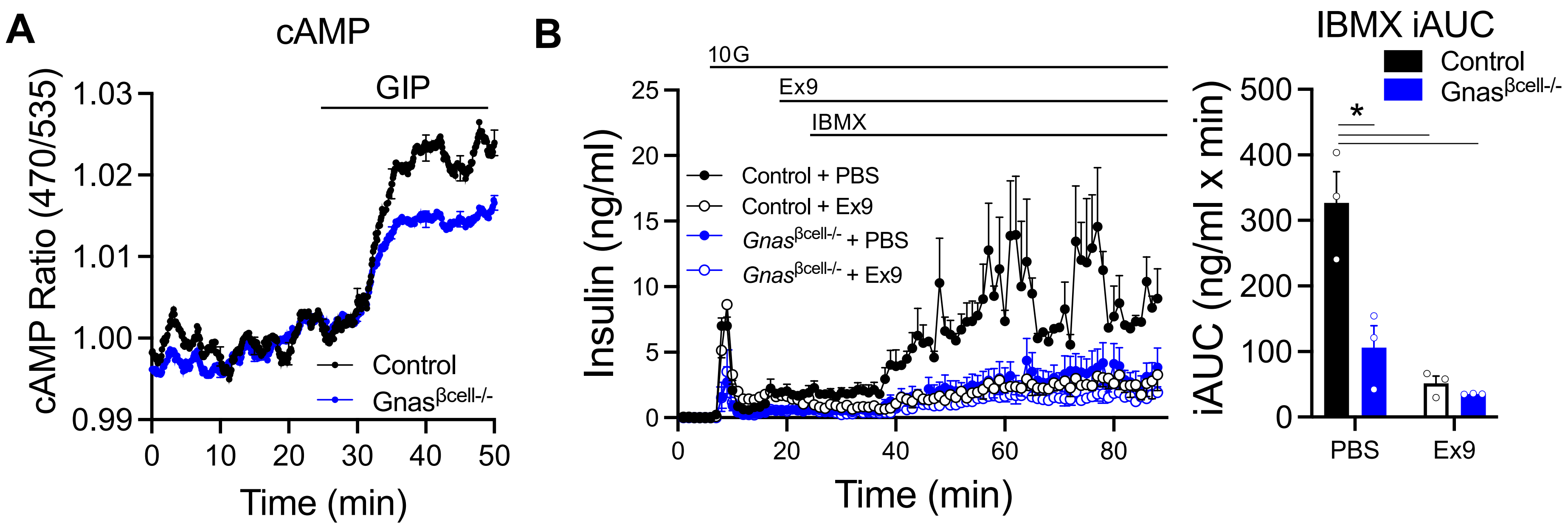

Supplemental Figure 4. Intracellular cAMP levels in β-cells imaged in live cells. (A) Real-time cellular cAMP levels in β-cells in response to GIP. (B) Insulin secretion during islet perifusion in response to high-glucose +/- Ex9 (1 uM) +/- IBMX. (n=3) (C) Insulin content in whole islets from control (n=4) and *Gnas*$^{\beta cell-/-}$ (n=8) mice. Data are shown as mean ± SEM.

Supplemental Figure 5

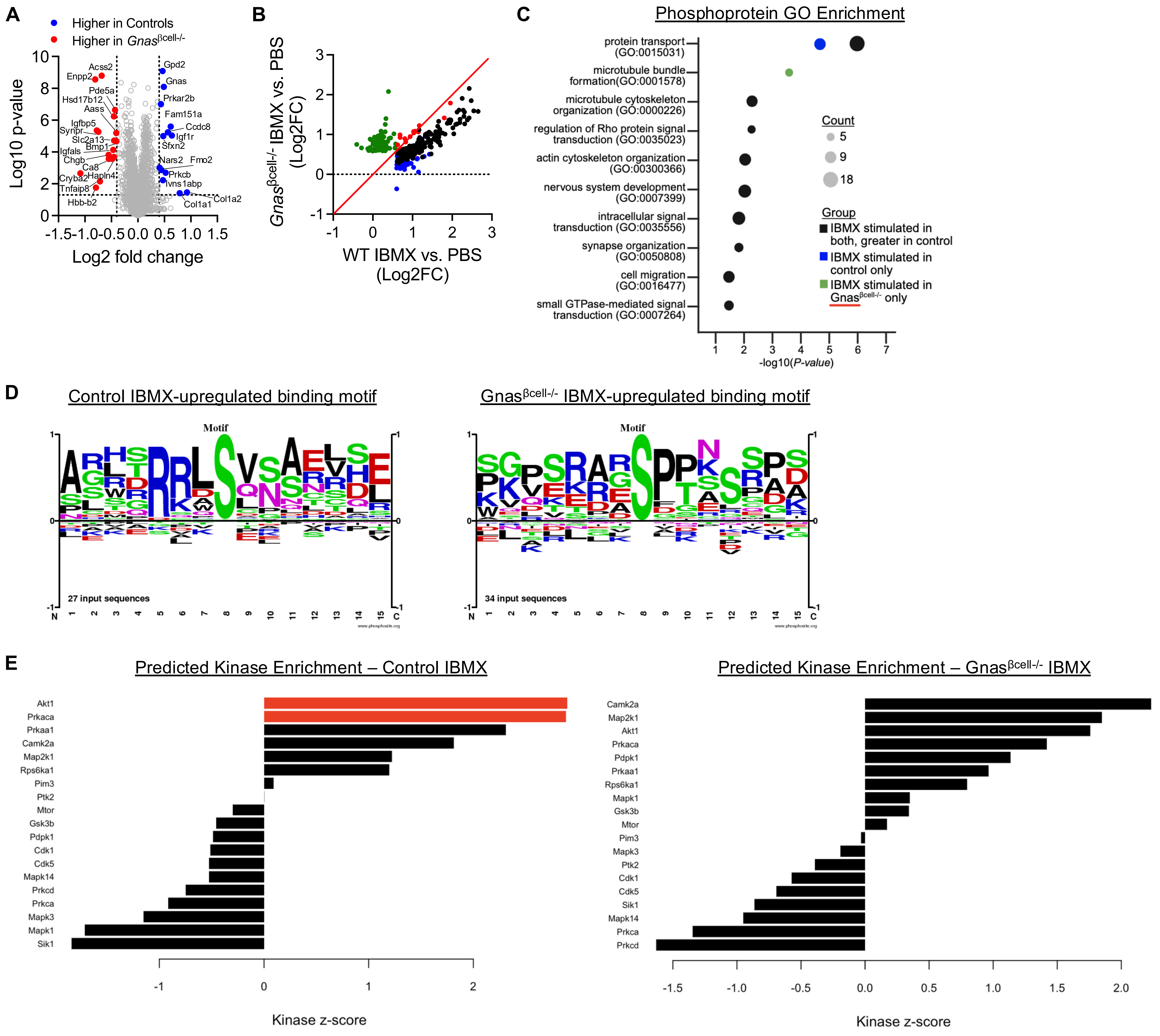

Supplemental Figure 5. *Gnas*$^{\beta cell-/-}$ islets have altered protein transport and impaired PKA-dependent signaling. (A) Volcano plots showing differences in protein levels between control and *Gnas*$^{\beta cell-/-}$ islets. Thresholds of ≥0.48 Log2fold change and ≥1.3 Log10 p-value are displayed. (B) Significantly upregulated phosphosites stimulated by IBMX in control and *Gnas*$^{\beta cell-/-}$ islets. Phosphosites significantly upregulated only in *Gnas*$^{\beta cell-/-}$ islets (green), increased to the same extent in controls and *Gnas*$^{\beta cell-/-}$ islets (red), increased to a greater extent in controls than *Gnas*$^{\beta cell-/-}$ islets (black) and significantly upregulated only in control islets (blue) are depicted. (C) GO Enrichment pathways identified using phosphorylated proteins displayed in (B). (D) Motif analysis using phosphosites significantly upregulated only in *Gnas*$^{\beta cell-/-}$ islets (green, B). (E) Motif analysis using phosphosites significantly upregulated only in control islets (blue, B) or only in *Gnas*$^{\beta cell-/-}$ islets. N=4 per group. (F) Kinase-substrate enrichment analysis bar plots for control (above) and *Gnas*$^{\beta cell-/-}$ (below) phosphoproteomics data. Kinases associated with an FDR-correct p-value less than 0.05 are highlighted in red. N=4 per group.

Supplemental Figure 6

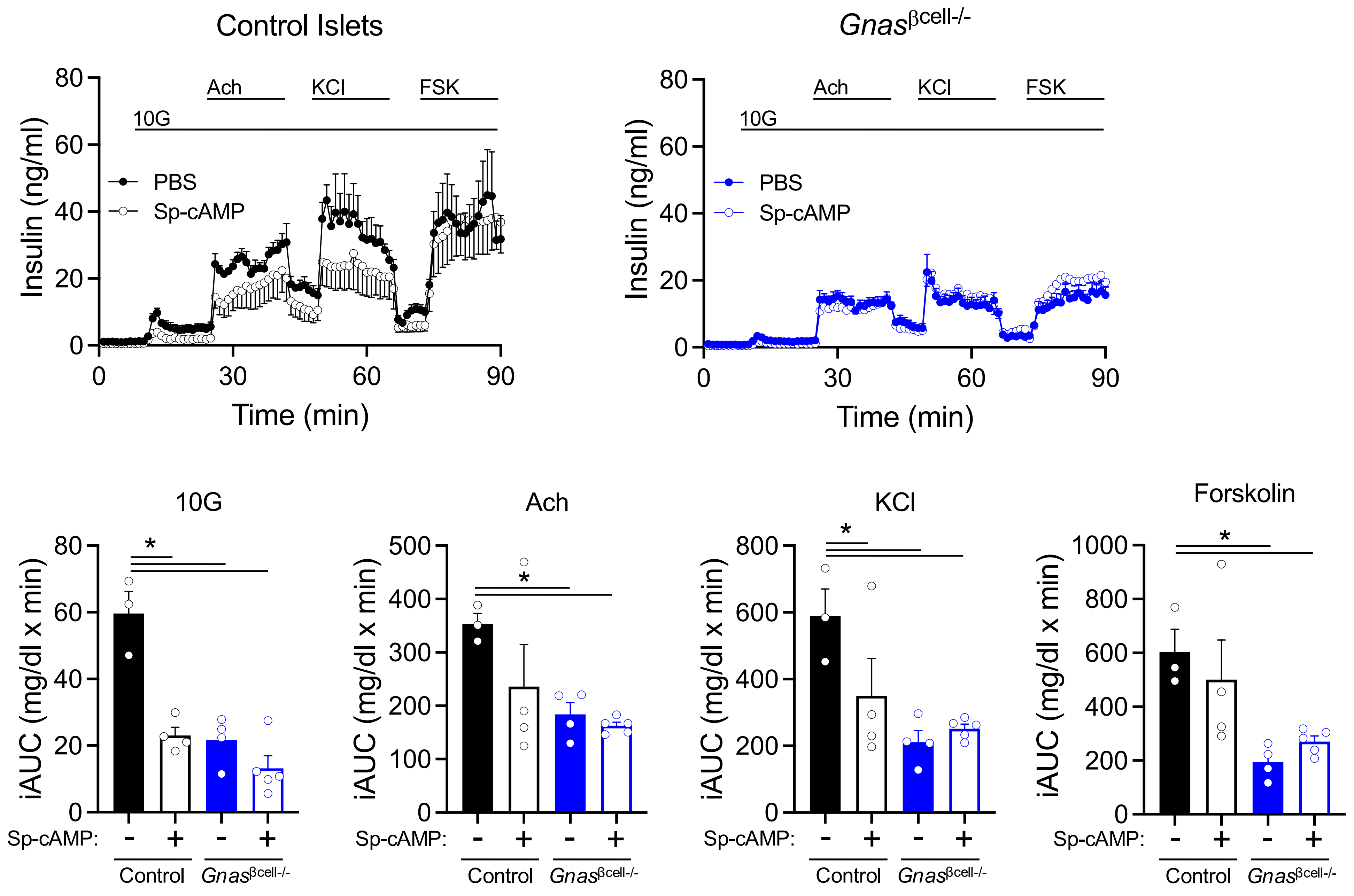

Supplemental Figure 6. Chronic treatment with a cAMP analog. Control and *Gnas*$^{\beta cell-/-}$ islets were isolated and treated in culture with Sp-8-BnT-cAMPS for 4 days. This was followed by a 24 hr washout period and a perifusion experiment. Data are shown as mean ± SEM. Data were analyzed by 2-way ANOVA. N=3,4,4,5.

Supplemental Figure 7

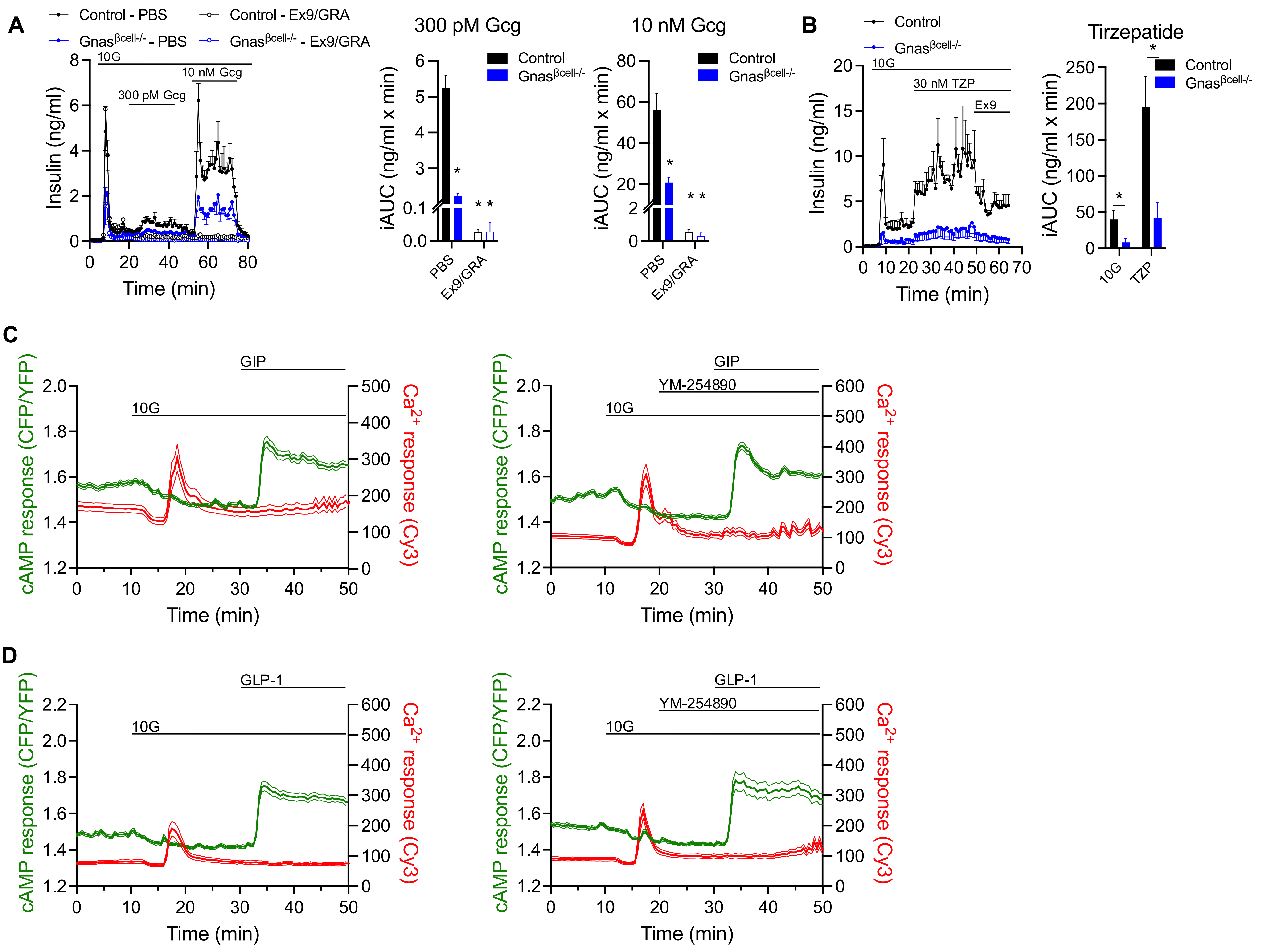

Supplemental Figure 7. Insulin secretion in response to glucagon or tirzepatide. (A) Insulin secretion in response to glucagon in the presence of absence of Ex9 and a glucagon receptor antagonist (GRA). (B) Insulin secretion in the response to tirzepatide. (C) cAMP and Ca2+ traces in wild-type islets in the presence of absence of YM-254890. Data are shown as mean +/- SEM, Data were analyzed by 2-way ANOVA. * - p<0.05 vs control or as indicated. N=3 for all groups (A,B). For panel C – GIP, control, n=69; GIP, YM-254890, n=64; GLP-1, control, n=33; GLP-1, YM-254789, n=37.
