# Supplementary material for: β Cell Gαs signaling is critical for physiological and pharmacological enhancement of insulin secretion
Source: J Clin Invest. 2025 Jun 17;135(16):e183741. doi: 10.1172/JCI183741 (PMC12352888; doi:10.1172/JCI183741)
Supplement: Unedited blot and gel images [file jci-135-183741-s198.pdf]

Uncropped/unedited gel image (Figure 3C)

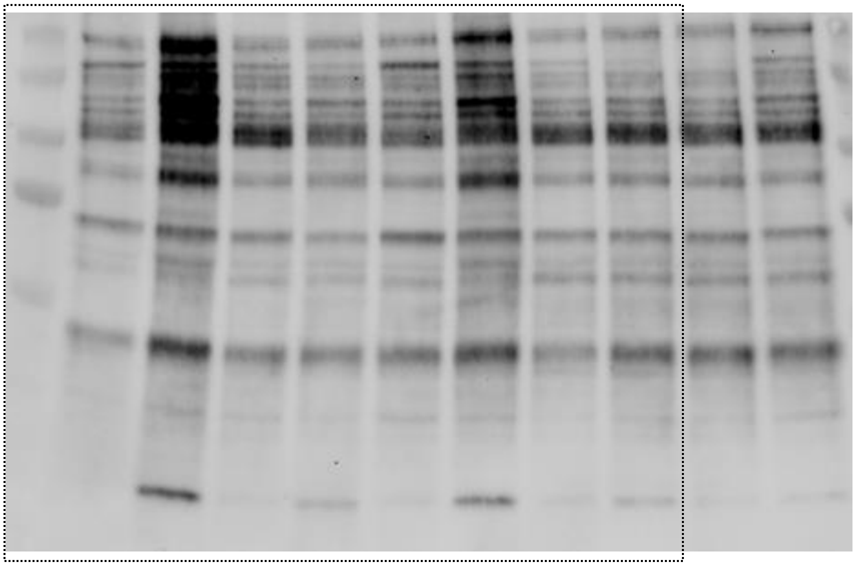

This is an unedited image of the gel used for Figure 3C. The dotted line indicates the cropped area used for Figure 3C. This is a PKA substrate blot, with a phospho-PKA substrate primary antibody (Cell Signaling, 9624, Rabbit mAb) and an anti-rabbit secondary (Cell Signaling, 7074). Images were developed with ECL substrate (Bio-Rad) and imaged in a ChemiDoc imager (Bio-Rad).
